# Serum Metabolomics Associating With Circulating MicroRNA Profiles Reveal the Role of miR-383-5p in Rat Hippocampus Under Simulated Microgravity

**DOI:** 10.3389/fphys.2020.00939

**Published:** 2020-08-18

**Authors:** Hongyu Zhang, Jian Chen, Hailong Wang, Xin Lu, Kai Li, Chao Yang, Feng Wu, Zihan Xu, Huan Nie, Bai Ding, Zhifeng Guo, Yu Li, Jinfu Wang, Yinghui Li, Zhongquan Dai

**Affiliations:** ^1^State Key Laboratory of Space Medicine Fundamentals and Application, China Astronaut Research and Training Center, Beijing, China; ^2^Institute of Cell and Developmental Biology, College of Life Sciences, Zhejiang University, Hangzhou, China; ^3^School of Life Sciences and Technology, Harbin Institute of Technology, Harbin, China

**Keywords:** microgravity, metabolome, miRNAome, miR-383-5p, aquaporin 4, hippocampus, space adaptation syndrome

## Abstract

Microgravity impacts various aspects of human health. Yet the mechanisms of spaceflight-induced health problems are not elucidated. Here, we mapped the fusion systemic analysis of the serum metabolome and the circulating microRNAome in a hindlimb unloading rat model to simulate microgravity. The response of serum metabolites and microRNAs to simulated microgravity was striking. Integrated pathway analysis of altered serum metabolites and target genes of the significantly altered circulating miRNAs with Integrated Molecular Pathway-Level Analysis (IMPaLA) software was mainly suggestive of modulation of neurofunctional signaling pathways. Particularly, we revealed significantly increased miR-383-5p and decreased aquaporin 4 (AQP4) in the hippocampus. Using rabies virus glycoprotein–modified exosomes, delivery of miR-383-5p inhibited the expression of AQP4 not only in rat C6 glioma cells *in vitro* but also in the hippocampus *in vivo*. Using bioinformatics to map the crosstalk between the circulating metabolome and miRNAome could offer opportunities to understand complex biological systems under microgravity. Our present results suggested that the change of miR-383-5p level and its regulation of target gene AQP4 was one of the potential molecular mechanisms of microgravity-induced cognitive impairment in the hippocampus.

## Introduction

Spaceflight impacts various aspects of the space travelers’ health, known as space adaptation syndrome, including bone loss, skeletal muscle atrophy, cardiovascular dysfunction, immune system dysregulation, and alterations in circadian rhythms and cognitive functions (Manzey and Lorenz, 1998; Grimm et al., 2016; Rea et al., 2016). Thus, there is a critical need to understand the mechanisms of how microgravity causes space adaptation syndrome and develop highly effective countermeasures to prevent these problems. Furthermore, understanding the mechanisms of spaceflight-induced space adaptation syndrome could help us to provide insight into the pathophysiology of diseases occurring on Earth (Nichols et al., 2006).

Many experimental methods or tools have been developed and used to study microgravity-induced pathophysiological changes. However, there remains a lack of systematic investigation on the microgravity-induced effect, which is the key information needed to ultimately unfold the mechanism behind microgravity-induced physiological alternations. Recently, current omics tools such as genomic, transcriptomic, proteomic, metabolomic, and epigenetic approaches, allowing large-scale, high-throughput analyses for the detection, identification, and functional investigation, have gained a lot of attention (Nichols et al., 2006; Rea et al., 2016; Schmidt et al., 2016; Kononikhin et al., 2017; Mao et al., 2018; Jin et al., 2019; Li et al., 2020). In particular, metabolomic and miRNAomic analyses in biofluids offer new methods for discovering biomarkers and molecular pathways underlying pathophysiological changes (Chen et al., 2016). Beheshti et al. (2018) identified 13 miRNAs which are common changes in the NASA GeneLab database and found they directly interacted with TGF-β pathway, which demonstrated the existence of the master regulator during the systemic response to microgravity and/or radiation.

In the present study, we first profiled and integrated the serum metabolome and miRNAome from rats undergoing simulated microgravity using hindlimb unloading (HU) method, aiming to probe the composition of serum metabolites and circulating microRNAs. Through multi-omics analysis, several differently regulated miRNAs found in serum were the master regulators mediating microgravity-induced effects in other tissues, especially miR-383-5p and its target gene water channel aquaporin 4 (AQP4) in hippocampus. Not only does AQP4 play an important role in regulating water balance in brain tissue (Papadopoulos et al., 2002) but also AQP4 in astrocytes plays an unanticipated role in cognitive function of the hippocampus (Skucas et al., 2011; Vandebroek and Yasui, 2020). Subsequent downstream *in vitro* and *in vivo* model was used to suggest miR-383-5p can affect the expression of AQP4 in the hippocampus. Therefore, multi-omics methods can help us to detect changes through systematically understanding gene and/or protein expression and molecular networks in various tissues, and unravel the mechanisms behind microgravity-induced problems and find effective countermeasures to spaceflight-induced alterations.

## Materials and Methods

All the experiments and processes in this study adhered to biosecurity and institutional safety procedures.

### Animal Experiments

The protocol of all animal experiments was approved by the Animal Care Committee of China Astronaut Research and Training Center. All the processes were according to the institutional guidelines for laboratory animals and adhered to standard biosecurity and institutional safety procedures. A total of 72 male Sprague-Dawley (SD) rats aged 7 weeks were purchased from the Animal Center of the Academy of Military Medical Sciences (Beijing, China) and housed at the Institutional Animal Care Facility of the China Astronaut Research and Training Center. SD rats have time to acclimatize to their new surroundings for 1 week separately before normal experiments, given free access to water and standard chow under a 12:12 h light/dark cycle.

For the simulated microgravity experiments, 60 male rats were randomly assigned to the control group (*n* = 30) or the hindlimb unloading treatment (HU) group for different days (*n* = 30). Mice were maintained one per cage. The HU manipulation was performed as Wronski described previously (Wronski and Morey-Holton, 1987) with minor modifications. Briefly, the rat was suspended by the tail attached to a chain hanging from a beam using a strip of adhesive surgical tape. The bodies of rats were at a 30° angle to the floor, with only the forelimbs touching the floor and allowed to freely move to food and water. Rats were anesthetized with 10% chloral hydrate (4 ml/kg) after hindlimb unloading for 14, 28, and 35 days. Blood was collected by cardiac puncture and centrifuged at 2000 × *g* for 10 min at 4°C, followed by 20 min high-speed centrifugation at 12,000 × *g* at 4°C to completely remove cell debris. The whole process was accomplished within 2 h and the supernatant serum was stored at −80°C until analysis. Hippocampus samples were frozen in liquid nitrogen for gene and protein expression analysis.

For the exosome injection experiments, 12 SD rats were intravenously injected with 1000 μg nerve-specific miR-383-5p-rich rabies virus glycoprotein (RVG) exosomes or unmodified vehicle exosomes (*n* = 6, each) for once. After 24 h, the rats were euthanized, then the hippocampus tissues were separated and frozen in liquid nitrogen for the assays of miR-383-5p, AQP4 mRNA, and protein levels assayed by RT-qPCR or Western blot as the following description.

### Gene Expression Analysis

Total RNA was extracted from rat hippocampus and glioma cell using standard Trizol-based protocols according to the manufacturer’s instructions. Then, 1 μg total RNA was used to synthesize cDNA with the PrimeScript RT reagent Kit (Takara, Dalian, China; code no. RR047A). The expression of interesting genes was detected by reverse transcription-quantitative polymerase chain reaction (RT-qPCR) using SYBR Premix Ex Taq II kit (Tli RNaseH Plus; Takara, Dalian, China; code no. RR820A) and the Roche LightCycler 96 system (Roche, Switzerland). Relative mRNA levels were calculated using the comparative Ct (cycle threshold) method and normalized to β-actin mRNA. All primers used for RT-qPCR are listed in Supplementary Table S1.

For serum miRNA detection, the extracting method was used as previously described (Chen et al., 2008). In brief, pipetted 200 μl serum was added with 600 μl RNAiso for small RNA (Takara). Cel-miR-39 was spike-in before RNA isolation to act as an additional quality control. Then miRNAs were extracted according to the manufacturer’s instructions, followed by reverse transcription with SYBR PrimeScript miRNA RT-PCR Kit (Takara, Dalian, China). qPCR was conducted in a total volume of 20 μl using SYBR Premix Ex Taq Kit (Takara) and the PCR cycling program included 95°C for 2 min, 40 cycles of 95°C for 10 s, 60°C for 20 s, and 72°C for 20 s; melting curve analysis was carried out at the end of cycling program. The qPCR was performed in triplicate on LightCycler 96 (Roche, Switzerland).

### miRNA Microarray

Serum miRNA microarrays were performed using three pooled control samples and four pooled HU samples (each pool was composed of four individual serum samples with the same volume). miRNA microarray was performed by Oebiotech (Shanghai, China). Briefly, total miRNA was quantified by the NanoDrop ND-2000 (Thermo Fisher Scientific, Rockford, United States) and the miRNA integrity was assessed using Agilent Bioanalyzer 2100 (Agilent Technologies, Santa Clara, CA, United States). The sample labeling, purification, microarray hybridization, and washing were performed based on the manufacturer’s standard protocols, recommended agents, and devices. After washing, the arrays (Agilent Rat miRNA 8^∗^60K, Design ID: 046066) were scanned with the Agilent Scanner G2505C. Feature Extraction software (version 10.7.1.1; Agilent Technologies) was used to analyze array images to get raw data. Next, GeneSpring software (version 12.5; Agilent Technologies) was employed to finish the basic analysis of the raw data. Differentially expressed miRNAs were then identified through fold change (≥1.5) as well as the *p* value (≤0.05) calculated by *t*-test.

### Western Blotting

Rat hippocampus tissue protein was prepared using RIPA buffer (50 mM Tris–HCl (pH 7.4), 150 mM NaCl, 1% NP-40, 1 mM EDTA, and 0.1% sodium dodecyl sulfate) containing protease inhibitor cocktail (Roche, Mannheim, Germany). Each sample was kept on ice for 30 min before centrifugation at 12,000 × *g* for 30 min at 4°C. The protein concentration of the supernatant was determined using a bicinchoninic acid protein assay kit according to the manufacturer’s instructions (Thermo Fisher Scientific). Proteins were subjected to 10% polyacrylamide gel electrophoresis and then transferred onto a polyvinylidene difluoride membrane (Millipore, Darmstadt, Germany). Membranes were blocked with 5% skim milk in Tris-buffered saline with 0.1% Tween 20 for 1 h and then incubated overnight with primary antibody diluted with 5% bovine serum albumin at 4°C. Antibodies specific for AQP4, DHPR, MAP2, BDNF, KCNH8, SLC1A2, Lamp1 (ABclonal Technology, China), CD63 (Abcam, United States), and TSG101 (Santa Cruz, United States) were used. β-Actin or GAPDH (Cell Signaling Technology, United States) served as a loading control. The samples were then incubated with the appropriate secondary antibodies conjugated to horseradish peroxidase (Cell Signaling Technology, United States). Signals were detected using enhanced luminescence (Applygen Technologies, China), and the intensity of protein bands was quantified using ImageJ software (NIH, Windows version).

### Serum Metabolomics

A 100-μl aliquot from each of 10 control and 10 HU-28 (HU for 28 days) serum samples were diluted with 300 μl of precooled (4°C) methanol–acetonitrile (v/v, 150 μl:150 μl) to remove protein. Leucine enkephalin (L-ENK, MW 555.62 Da, 200 pg/μl in acetonitrile/water 50:50) was used as a lock mass. Quality control (QC) samples were prepared by combining equal aliquots of serum and processed in the same way as the analytical samples. The QC samples were injected regularly (every 10 samples) throughout the run to monitor the stability of the LC/MS platform.

Unbiased metabolic profiling was operated in positive electrospray ionization (ESI+) and negative electrospray ionization (ESI−) mode, using ultra-performance liquid chromatography (Waters Corporation, Milford, MA, United States) coupled to quadrupole-time-of-flight mass spectrometry (UPLC/Q-TOF MS), with a Waters Acquity BEH C18 column (2.1 × 100 mm, 1.7 μm) and an Agilent 6520 Q-TOF LC/MS (Agilent Technologies).

The metabolomics profiling data from UPLC/MS was imported into MassHunter Qualitative Analysis software (Agilent Technologies) to extract metabolic features, using the Molecular Feature Extraction Algorithm. The resultant metabolic features were submitted to the XCMS Online program^1^ to detect metabolic features and match chromatographs, and default values were used for all parameter settings in XCMS (Rinehart et al., 2014). After metabolic peak detection and alignment, a total of 4983 or 4406 signals of metabolites from serum were identified under ESI+ or ESI− conditions. Before multivariate analysis, the outer datasets were further trimmed using Microsoft Excel (version 2007; Microsoft), where the peak areas were normalized by sum, the resultant ion features were arranged in a two-dimensional matrix consisting of an arbitrary peak index (RT–m/z pair), and the peak area was normalized for further analysis.

Principal component analysis (PCA) was performed with SIMCA-P software (version 14.1; Umetrics, Sweden), and the corresponding R2X, R2Y, and Q2Y values were computed for multivariate statistical analysis. In the present study, discriminating variables were selected based on an importance in the projection (VIP) value greater than 1, jackknife CIs were calculated, and raw data were plotted using the orthogonal partial least squares–discriminant analysis (O2PLS-DA) model. Finally, potential discriminating metabolites were chosen with adjusted *p* values less than 0.05.

Extracted m/z values from XCMS were submitted to online databases (HMDB, KEGG)^2,^^3^ for database searching. Additionally, MS/MS data were referenced to validate metabolites and exclude peaks caused by neutral losses, isotopes, and productions.

Metabolic pathway analysis was carried out to identify the affected metabolic pathway using the online program IMPaLA^4^ (Kamburov et al., 2011).

### Cell Culture

The human embryonic kidney cell line 293 (HEK293), C2C12, and SH-SY5Y cell lines were cultured in high-glucose DMEM, supplemented with 10% FBS and antibiotics. The rat C6 glioma cells were cultured in F10 medium, supplemented with 2.5% FBS, 10% horse serum, and 1% antibiotics penicillin and streptomycin (Gibco, United States). All cells were incubated at 37°C in 5% CO_2_.

### Construction of pGL3-AQP4-3UTR

The cDNA from hippocampus RNA was used to amplify the 3’-untranslated regions by Pfu Ultra High-Fidelity DNA Polymerase (Takara, Dalian, China) and its primers (Supplementary Table S1). The fragment of 1996 bp was inserted into pGL3-promoter by *Xba*I digestion. The constructed vector was verified by DNA sequencing.

### Preparation of Nerve-Specific Targeting Exosomes

The miR-383-5p mimics, inhibitor, and shRNA vector were synthesized by GenePharma (China). As previously described (El-Andaloussi et al., 2012), the RVG-derived peptide was fused to upstream of Lamp2b, which was cloned from C2C12 cells using the primers Lamp2b-5 and Lamp2b-3 (Supplementary Table S1), then inserted into pEGFP-C1 plasmid between *Xho*I and BspE1 site, named pRVG-Lamp2b. A total of 293 cells were co-transfected with miR-383-5p mimics shRNA plasmid and the constructed pRVG-Lamp2b plasmid using Lipofectamine 3000 (Invitrogen, United States), when the cells reached approximately 70–80% confluence. The cell culture medium was harvested 48 h after transfection for exosome isolation using an exosome isolation kit (Invitrogen, United States). The purified pellet was resuspended in PBS and incubated in 5 μg/ml CellMask (Invitrogen, United States) fluorescent dye for 15 min at 37°C. Then the labeled exosomes were harvested as previously described and resuspended in PBS or saline before use.

### Transmission Electron Microscopy Assay

The purified exosomes were resuspended in PBS and fixed with 2% paraformaldehyde for 30 min at room temperature. The mixture (30 μl) was then dropped onto electron microscopy grids which had been pretreated with UV light to reduce static electricity. After drying for 30 min, exosomes were stained twice (6 min each) with 1% uranyl acetate. The dried grids were examined using a JEM 1200EX (JEOL, Japan) transmission electron microscope (TEM) at a voltage of 120 kV.

### Nanoparticle Tracking Analysis (NTA)

Nanoparticle tracking analysis was performed with the Zetasizer Nano S system (Malvern, United Kingdom) using purified exosomes resuspended in saline. The system focuses a laser beam through a suspension of the particle of interest. The results are visualized by light scattering.

### Confocal Microscopy Analysis

Fluorescence-labeled exosomes (50 μg for 10^6^ cells) were incubated with the SH-SY5Y cells and C6 cells, respectively. After 8 h, the cells were washed, fixed, and observed under a confocal microscope (TCS SP5; Leica, Germany).

### Flow Cytometric Analysis

The labeled exosomes were incubated with C6 cells for 1 h. Afterward, cells were washed two times with PBS and resuspended in 1 ml PBS. The RVG exosomes binding to C6 cells were detected using a flow cytometer (Becton Dickinson, United States) and analyzed by the Flow Jo software (Tree Star, United States).

### Data Analysis

Expression levels of the serum miRNA and relative genes were determined using the relative quantification method (2^–Δ^ ^Δ^ ^Ct^) with the selected reference gene miR-25-3p for miRNA and β-actin for mRNA. All generated quantitative data were presented as mean ± SD and differences were analyzed with one-way ANOVA or two-way ANOVA followed by Tukey’s honestly significant difference *post hoc* multiple-comparison test as appropriate. Statistical analysis was performed using SPSS (version 19.0; SPSS, United States). *P* value < 0.05 was considered as statistically significant difference (^∗^*p* < 0.05, ^∗∗^*p* < 0.01).

## Results

### Simulated Microgravity Alters Serum Metabolite Profiles in HU Rats

In the present study, we used HU model to investigate the effects of microgravity on the body. Food intakes of the HU-28 rats were not affected by hindlimb unloading which is consistent with the results previously reported (Feng et al., 2016). However, the level of bone mineral density, bone volume rate, and trabecular number of the HU-28 rats were substantially reduced by 44, 46, and 40%, respectively, whereas trabecular separation was significantly increased by 78% relative to baseline (*n* = 6, *p* < 0.001). Micro-computed tomography (MicroCT) results showed that the rats had significant osteoporosis after 28 days of hindlimb unloading and indicates that the HU model could be used to simulate the space microgravity (Chen et al., 2016). To better understand the mechanism of whole-body metabolism system disorders in rats under simulated microgravity (SMG), we first explored serum metabolomics using an untargeted metabolic profiling method.

**FIGURE 1:**
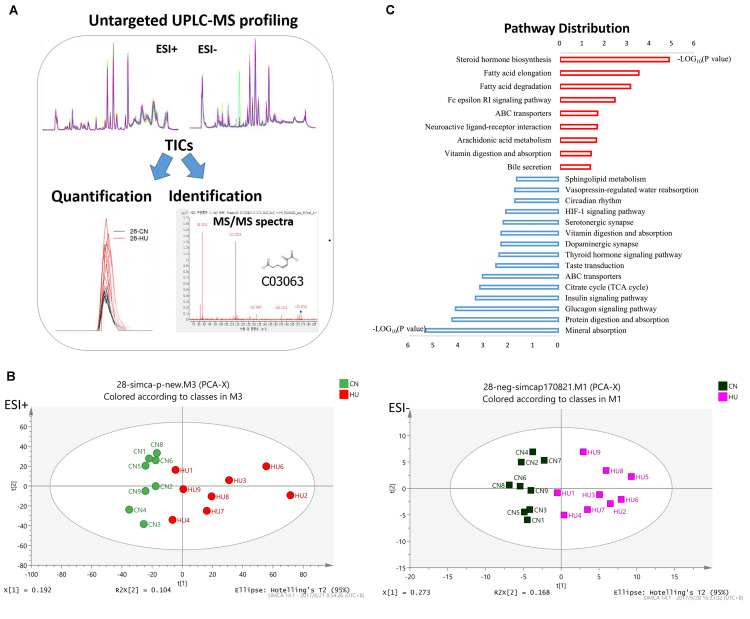
Serum metabolomics reveals different metabolic profiles between control and HU rats. **(A)** Metabolomic workflow. **(B)** PCA plot of scores separating HU-28 (red in ESI+ and pink in ESI–) and control (green in ESI+ and black in ESI–) serum samples. **(C)** Abundance of discriminating metabolites in pathways according to KEGG analysis. The red column or blue column represents an increase or decrease pathway, respectively.

Ultra-performance liquid chromatography–mass spectrometry (UPLC-MS) analysis was performed to determine relative levels of metabolites among control and HU-28 group samples (Figure 1A). In total, 4983 or 4406 signals of metabolites from serum were identified under electrospray ionization (ESI+) or negative electrospray ionization (ESI−) conditions. Then, datasets were subjected to supervised multivariate analysis to elucidate any metabolomics pattern that could differentiate between HU and control group compounds. The plot of the PCA scores showed that control and HU-28 individuals were divided into two distinct clusters based on the first principal component in both ESI+ and ESI− models (Figure 1B). Using O2PLS-DA, these two groups could be further divided. The high accuracy (ESI+ : Q2 = 0.699 or ESI−: Q2 = 0.931) reflected the fact that individual differences of rats had little impact on the experiment, indicating that environmental conditions of HU were well controlled in the present study (Supplementary Figure S1A). Moreover, the interpretative ability of the O2PLS-DA model was validated using the permutation test with 200 iterations. All Q2 values (ESI+ : Q2 = −0.0477 or ESI−: Q2 = −0.607) in blue on the left were lower than the original points in green on the right and the blue line of Q2 points intersects the vertical axis below zero, indicating that the original model was valid (Supplementary Figure S1B). Finally, a total of 135 metabolites that displayed significantly different levels between HU-28 and control groups were screened (*p* < 0.05; VIP > 1; Supplementary Table S2 for UPLC-MS).

To deeply study these discriminating metabolites, we explored the functional enrichment of metabolites using the Kyoto Encyclopedia of Genes and Genomes (KEGG) pathway database. As shown in Figure 1C, the pathways of upregulated metabolites mainly included steroid hormone biosynthesis, fatty acid metabolism, neuroactive ligand–receptor interaction, arachidonic acid metabolism, and vitamin digestion and absorption, however, the downregulated pathways were mainly related to mineral absorption, protein digestion and absorption, glucagon signaling pathway, insulin signaling pathway, tricarboxylic acid cycle, thyroid hormone signaling pathway, dopaminergic synapse, serotonergic synapse, hypoxia-inducible factor 1 signaling pathway, circadian rhythm, vasopressin-regulated water reabsorption, and sphingolipid metabolism processes. The results suggested that SMG could induce the obvious changes in energy metabolism and nervous system metabolism of the body.

### SMG Profoundly Influences the Global Circulating miRNA Expression in Serum

**FIGURE 2:**
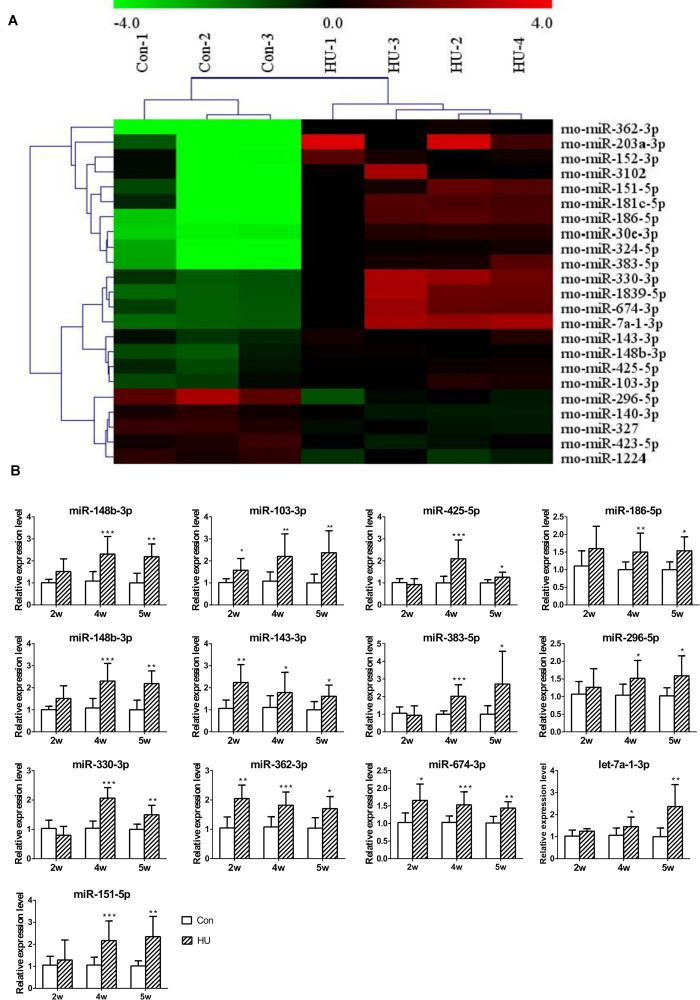
Analysis of differential expression of serum miRNAs in HU rats. **(A)** miRNA microarray was performed to analyze the expression of miRNAs in the serum of HU rats; 18 upregulated and 5 downregulated miRNAs were found in the serum of HU rats compared with control groups; the cutoff was a fold change ≥ 1.5 and *p* value < 0.05. **(B)** 13 upregulated miRNAs were confirmed by qPCR which were normalized to miR-25-3p (*n* = 12). *p < 0.05, **p < 0.01, ***p < 0.001, HU vs. Con.

Next, to investigate the influence of SMG on the circulating miRNAome, serum miRNA microarrays were performed. The threshold set for significantly changed genes was a fold change ≥1.5 and *p* value ≤ 0.05. As shown in Figure 2A, 18 miRNAs were upregulated and 5 miRNAs were downregulated in the serum of HU rats compared with the control groups. Then, RT-qPCR was performed to validate the differential expression of genes in an independent set of 40 rat serum samples. By normalizing to miR-25-3p, a reliable reference gene that we previously screened and validated (Chen et al., 2016), the results showed that 13 miRNAs (miR-148b-3p, miR-103-3p, miR-425-5p, miR-186-5p, miR-30e-3p, miR-143-3p, miR-383-5p, miR-296-5p, miR-330-3p, miR-362-3p, miR-674-3p, let-7-1-3p, and miR-151-5p) were upregulated from microarrays in the rat serum after HU for 4 and 5 weeks (Figure 2B).

To gain insight into miRNA-mediated pathways affected by SMG, we predicted the mRNA targets of these altered circulating miRNAs with two commonly used databases (TargetScan and miRanda) and retained only 263 shared target genes predicted by two databases for further analysis (Supplementary Table S3). Using DAVID software,^5^ the predicted biological progresses were ranked according to the *p* value and Benjamini and found that nervous system development was likely to be highly influenced under the SMG (Table 1). Furthermore, the results of cluster analysis of target gene-mediated pathways were involved in multiple neurological related pathways, as well as retrograde endocannabinoid signaling, glutamatergic synapse, and circadian entrainment pathways, suggesting that SMG may modulate neurological activity through the miRNA-mediated post-transcriptional regulation (Table 2). Besides, the results of tissue expression analysis of target genes had shown mainly related to brain and hippocampus (Table 3). It was suggested that SMG-altered serum miRNA could be associated with the nervous system, especially the biological function of hippocampus.

**TABLE 1:** Biological process analysis of the target genes of differential expression microRNAs using DAVID bioinformatics resources.

**Category**	**Term**	**Count**	***P* value**	**Benjamini**
GOTERM_BP_DIRECT	Nervous system development	13	2.00E−05	3.30E−02
GOTERM_BP_DIRECT	Cellular response to organic cyclic compound	9	2.80E−04	2.10E−01
GOTERM_BP_DIRECT	Inner ear development	7	3.10E−04	1.60E−01
GOTERM_BP_DIRECT	Cellular response to cAMP	7	6.50E−04	2.40E−01
GOTERM_BP_DIRECT	Brain development	13	1.00E−03	2.90E−01
GOTERM_BP_DIRECT	Cellular response to dexamethasone stimulus	6	1.30E−03	3.00E−01
GOTERM_BP_DIRECT	Regulation of growth	5	1.30E−03	2.70E−01
GOTERM_BP_DIRECT	Positive regulation of transcription from RNA Polymerase II promoter	27	1.80E−03	3.10E−01
GOTERM_BP_DIRECT	Negative regulation of translation	6	1.80E−03	2.90E−01
GOTERM_BP_DIRECT	Negative regulation of gene expression	10	2.60E−03	3.60E−01
GOTERM_BP_DIRECT	Negative regulation of neuron differentiation	6	3.20E−03	3.90E−01
GOTERM_BP_DIRECT	Transcription from RNA polymerase II promoter	14	3.50E−03	3.90E−01
GOTERM_BP_DIRECT	Response to nutrient levels	7	4.40E−03	4.40E−01
GOTERM_BP_DIRECT	Response to endoplasmic reticulum stress	6	5.30E−03	4.80E−01
GOTERM_BP_DIRECT	Positive regulation of dendritic spine development	4	7.10E−03	5.50E−01
GOTERM_BP_DIRECT	Cellular response to glucose stimulus	6	7.50E−03	5.50E−01
GOTERM_BP_DIRECT	RNA splicing	6	8.60E−03	5.80E−01

**TABLE 2:** KEGG pathway analysis of the target genes of differential expression microRNAs using DAVID bioinformatics resources.

**Annotation cluster 1**	**Enrichment score: 1.6**	**Count**	***P* value**	**Benjamini**
KEGG_PATHWAY	Retrograde endocannabinoid signaling	7	3.70E−03	1.10E−01
KEGG_PATHWAY	Glutamatergic synapse	6	2.60E−02	2.40E−01
KEGG_PATHWAY	Circadian entrainment	4	1.70E−01	5.90E−01

**Annotation cluster 1**	**Enrichment score: 1.43**	**Count**	***P* value**	**Benjamini**

KEGG_PATHWAY	Adrenergic signaling in cardiomyocytes	9	1.20E−03	2.00E−01
KEGG_PATHWAY	cAMP signaling pathway	10	2.00E−03	1.70E−01
KEGG_PATHWAY	Amphetamine addiction	6	2.30E−03	1.30E−01
KEGG_PATHWAY	Long-term potentiation	6	2.40E−03	1.10E−01
KEGG_PATHWAY	Dopaminergic synapse	8	2.60E−03	9.20E−02
KEGG_PATHWAY	Renin secretion	5	1.60E−02	2.80E−01
KEGG_PATHWAY	Oxytocin signaling pathway	7	2.90E−02	2.40E−01
KEGG_PATHWAY	Alcoholism	7	4.30E−02	3.00E−01
KEGG_PATHWAY	Insulin signaling pathway	6	5.30E−02	3.40E−01
KEGG_PATHWAY	cGMP–PKG signaling pathway	6	1.00E−01	5.00E−01
KEGG_PATHWAY	Aldosterone synthesis and secretion	4	1.20E−01	5.60E−01
KEGG_PATHWAY	Platelet activation	5	1.30E−01	5.60E−01
KEGG_PATHWAY	Circadian entrainment	4	1.70E−01	5.90E−01
KEGG_PATHWAY	Melanogenesis	4	1.70E−01	5.90E−01
KEGG_PATHWAY	Inflammatory mediator regulation of TRP channels	4	2.30E−01	6.80E−01
KEGG_PATHWAY	Vascular smooth muscle contraction	4	2.60E−01	6.70E−01
KEGG_PATHWAY	Estrogen signaling pathway	3	4.10E−01	7.80E−01
KEGG_PATHWAY	Cholinergic synapse	3	4.90E−01	8.40E−01

**TABLE 3:** Tissue-specific analysis of the target genes of differential expression microRNAs using DAVID bioinformatics resources.

**Category**	**Term**	**Count**	***P* value**	**Benjamini**
UP_TISSUE	Brain	69	7.50E−04	4.00E−02
UP_TISSUE	Hippocampus	23	1.00E−03	2.80E−02
UP_TISSUE	Heart	27	7.80E−03	1.30E−01
UP_TISSUE	Lung	24	4.60E−02	4.70E−01
UP_TISSUE	Forebrain	3	4.90E−02	4.20E−01

### miRNAome Analysis Associated With the Metabolic Phenotype Under SMG

**FIGURE 3:**
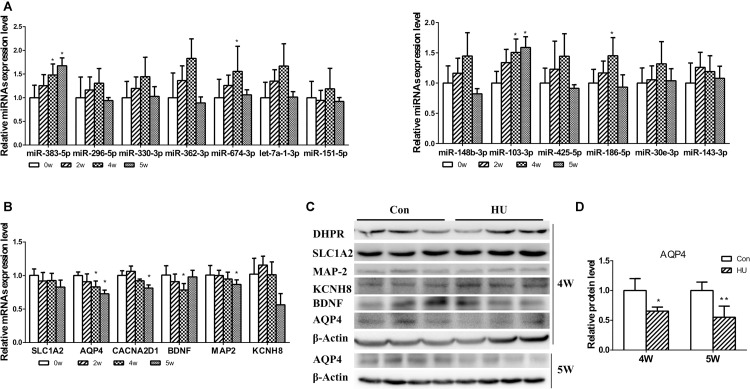
Analysis of differential expression miRNAs and target genes in the hippocampus. **(A)** 13 upregulated serum miRNAs were detected in the hippocampus after rat HU for 0, 2, 4, and 5 weeks; miR-103-3p and miR-383-5p showed significant upregulation (*n* = 5), *p < 0.05. **(B)** The mRNA expression levels of predicted target genes were detected in the hippocampus after rat HU for 0, 2, 4, and 5 weeks; AQP4 showed significant downregulation (*n* = 5), *p < 0.05. **(C)** The protein expression levels of predicted target genes were detected in the hippocampus after rat HU; AQP4 showed significantly downregulation after rat HU for 4 or 5 weeks (*n* = 3), The samples derive from the same experiment and the blots were processed in parallel, *p < 0.05. **(D)** Relative expression levels of AQP4 were normalized to β-actin (*n* = 3), *p < 0.05.

Following the analysis of predicted miRNA-targeted pathways, we investigated the joint analysis between the circulating miRNAome and the serum metabolomes. Using the web software called Integrated Molecular Pathway-Level Analysis (IMPaLA)^6^ for the combined analysis of gene/protein and metabolite datasets, we found that the top shared pathways (Sudden Infant Death syndrome Susceptibility Pathways, G Protein Signaling Pathways, Dopaminergic synapse, Sphingosine 1-phosphate pathway, Vasopressin-regulated water reabsorption, etc.) were mainly related to nervous system function (Table 4). Then, 13 upregulated circulating miRNAs were validated at different HU time points (0, 2, 4, and 5 weeks) (Figure 3A). It was only found that the levels of miR-103-3p and miR-383-5p showed significant upregulation in the hippocampus of HU rats. Therefore, the predicted target genes Excitatory amino acid transporter 2 (SLC1A2), Voltage-dependent calcium channel subunit alpha-2/delta-1 (CACNA2D1), Brain-derived neurotrophic factor (BDNF) of miR-103-3p and microtubule-associated protein 2 (MAP2), AQP4, and Potassium voltage-gated channel subfamily H member 8 (KCNH8) of miR-383-5p were selected for further investigation through rational analysis and screening of top shared pathways. Subsequently, the mRNA and protein expression levels of these selected distinctive sets of predicted target genes were detected in the hippocampus of HU rats at different time points. The results showed that the mRNA expression level of AQP4 was gradually significantly downregulated with the extended HU time (Figure 3B). Similarly, only the protein expression level of AQP4 from the selected predicted target genes was significantly reduced after rat HU for 4 and 5 weeks (Figures 3C,D). The mRNA and protein levels of other target genes had no obvious changes after rat HU (Figures 3B,C). These results suggested AQP4 may be the target gene of miR-383-5p.

**TABLE 4:** Correlated analysis between microRNAome and metabolic profiles using IMPaLA web tool.

**Pathway name**	**Overlapping genes**	**P genes**	**Metabolites**	**P metabolites**	**P joint**
SIDS susceptibility pathways	MAP2;YWHAB;CREBBP;YWHAH;BDNF; ADCYAP1;AQP4;GABRA1;NR3C1;REST	0.00016	C00079;C00078	0.00465	1.12E−05
G protein signaling pathways	AKAP6;CALM1;AKAP1;AKAP2;GNAI3; AKAP7;GNAZ	0.000467	C00575	0.0319	0.00018
Dopaminergic synapse	CALM1;PPP2R3C;CACNA1C;PPP1CB; GRIA3;GNAI3;CREB3L2;PPP1CC	0.00076	C00575;C05587	0.00396	4.13E−05
Oocyte meiosis	CALM1;SKP1;YWHAB;YWHAH;IGF1; PPP1CB;PPP1CC	0.00205	C00575	0.0319	0.000694
Sphingosine 1-phosphate (S1P) pathway	GNAZ;GNAI3;S1PR1	0.00417	C00018;C01120	0.000621	3.59E−05
Vasopressin-regulated water reabsorption	AQP4;CREB3L2;RAB11B;DYNC1LI2	0.00497	C00575	0.0161	0.000833
Adaptive immune system	CALM1;CDKN1B;RNF34;SKP1;YWHAB; PDIA3;UBE2D3;HSPA5;BTLA;WWP1; TRPC1;RAP1B;ARF1;UBE2B; CTSB;SH3KBP1;ENAH	0.007	C00188;C00183; C00079;C00078; C00575;C00135	1.65E−06	2.22E−07
FoxO signaling pathway	CDKN1B;CREBBP;S1PR1;GADD45A; IGF1;PRKAG2	0.0152	C00031	0.0397	0.00509
IL2 signaling events mediated by PI3K	CALM1;IL2;RPS6KB1	0.0161	C00575	0.0475	0.00626
Endochondral ossification	COL2A1;CALM1;PTH;IGF1	0.0173	C00575	0.0161	0.00256
Insulin signaling pathway	CALM1;RPS6KB1;EIF4E;PPP1CB; PPP1CC;PRKAG2	0.0207	C00031;C00575	0.000375	9.88E−05
Rap1 signaling	YWHAB;RAP1B	0.0257	C00575	0.0475	0.0094
Amino acid synthesis and interconversion (transamination)	GLUD1;PSPH	0.0288	C00077;C00018; C00940	0.00202	0.000626
Insulin secretion	CREB3L2;RIMS2;ADCYAP1;CACNA1C	0.0453	C00031;C00575	0.00465	0.00199
MAPK signaling pathway	MAP3K12;HSPA5;GADD45A;BDNF; RAP1B;PPM1B	0.0454	C00575	0.00806	0.00327

### miR-383-5p Changes Are Responsible for Altered AQP4 Expression

**FIGURE 4:**
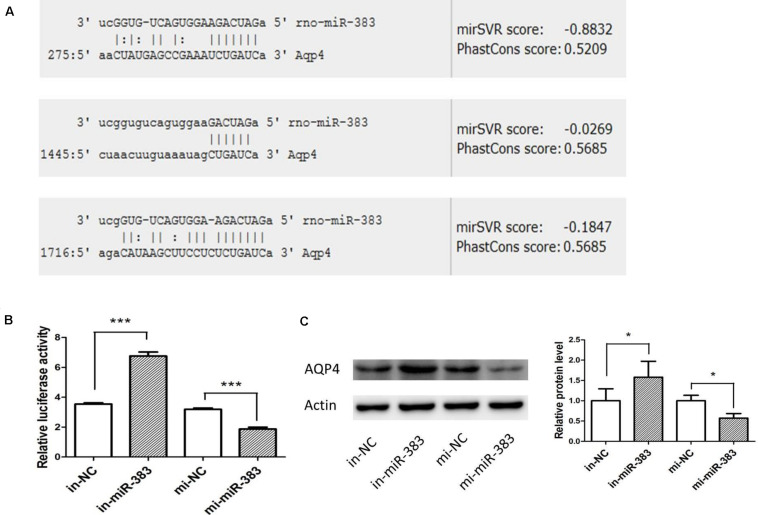
miR-383-5p directly targets AQP4 in glioma cells. **(A)** Sequence alignment of rat miR-383-5p with 3′-UTR of AQP4. The seed sequences of miR-383-5p (up) and 3′-UTR of AQP4 (down) are complementary. **(B)** The effect of mimics-miR-383-5p, inhibitor-miR-383-5p, and NC on luciferase activity in C6 cells transfected with the AQP4 3′-UTR reporter (*n* = 3), ***p < 0.001. **(C)** Effect of mimics miR-383-5p (mi-miR-383), inhibitor miR-383-5p (in-miR-383), and NC (mi-NC or in-NC) on AQP4 protein level by western blot analysis (*n* = 4). The samples derive from the same experiment and the blots were processed in parallel, *p < 0.05.

Using miRanda-mirSVR software, we found there were three potential target binding sites of miR-383-5p in the 3′ untranslated regions (UTRs) of rat AQP4 (Figure 4A). To verify whether miR-383-5p directly binds to the 3′ UTR of AQP4, the sequence of AQP4-3′ UTR containing three binding sites by PCR was cloned and inserted downstream the luciferase reporter gene in pGL3-promoter vector, named pGl3-AQP4-3UTR. The relative luciferase activity was markedly increased or decreased in C6 cells after cotransfection this constructed vector/phRL-TK with miR-383-5p inhibitor or its mimic, respectively (Figure 4B), compared with their negative control group. A similar result was obtained in the detection of AQP4 protein expression by western blot (Figure 4C). Therefore, our results suggested that AQP4 was a direct target of miR-383-5p in C6 cells.

### Exosome-Mediated Delivery of miR-383-5p Suppressed AQP4 Expression *in vitro* and *in vivo*

**FIGURE 5:**
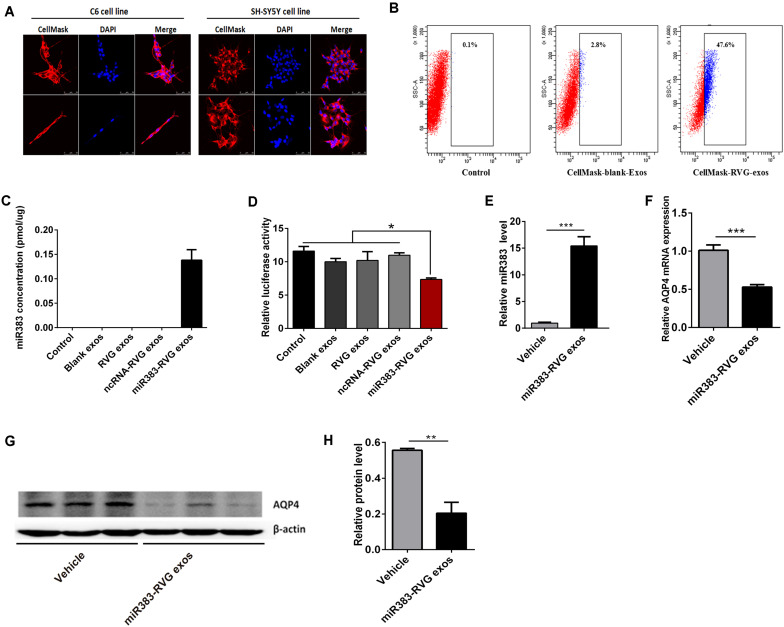
Exosome-mediated delivery of miR-383-5p suppressed AQP4 expression *in vitro* and *in vivo*. **(A)** Flow cytometric analysis of RVG exosomes binding to C6 cells. Exosomes purified from HEK 293 cells were labeled with CellMask and incubated with C6 for 1 h. CellMask-labeled RVG exosomes showed significantly higher binding compared with blank exosomes. No binding was observed in the control (without the addition of exosomes). **(B)** Monitoring of HEK293-derived exosome uptake *in vitro* into C6 and SH-SY5Y cells at 8 h after exosome treatment. Exosomes were labeled with CellMask (red) and cell nuclei were stained with DAPI (blue). Scale bars = 50 μm. **(C)** Absolute RT-qPCR analysis of miR-383-5p levels in various exosomes. **(D)** Relative luciferase activity was measured in C6 cells incubated with various exosomes after transfected with the pGL3 plasmid including the 3′-UTR of AQP4. **(E)** RT-qPCR analysis of miR383 concentration in the hippocampus of rats treated with saline and miR383 in RVG exosome. **(F)** RT-qPCR analysis of AQP4 mRNA levels in the hippocampus of rat treatment as described in panel **(E)**. **(G)** Western blot analysis of AQP4 protein levels in the hippocampus of rat treated as described in panel **(E)**. The samples derive from the same experiment and the blots were processed in parallel. **(H)** Quantification of the AQP4 protein levels in panel **(G)**. *n* = 6, *p < 0.05; **p < 0.01.

Using RVG-modified exosomes, we tried to verify whether the delivery of miR-383-5p could alter the AQP4 expression level *in vitro* and *in vivo*. First, human embryonic kidney 293 (HEK 293) were co-transfected with pRVG-Lamp2b and miR-383-5p plasmids to generated miR-383-5p-rich RVG exosome. The characterization of the pellets purified from co-transfected HEK293 supernatant was detailed as a classical exosome. The physical property of the modified exosomes did not appear to be affected by TEM analysis (Supplementary Figure S2A) and showed a single peak and have a mode size of 78.8 nm by NTA (Supplementary Figure S2B), which conformed to the sizes of exosomes reported in the literature (40–100 nm). Western blot analysis of exosomal markers CD63, Lysosomal-associated membrane protein 1 (Lamp1), and heat shock protein 70 (HSP70) further confirmed the property of the purified modified exosomes (Supplementary Figure S2C). Second, the HEK 293–derived RVG exosomes were labeled by CellMask dye and incubated with C6 cells or human neuroblastoma SH-SY5Y cells. The modified RVG exosomes showed highly efficient targeting neural cell lines as demonstrated by confocal imaging and flow cytometry (Figures 5A,B). Third, absolute quantification PCR results showed this RVG exosome was enriched with miR-383-5p (Figure 5C). Using a dual-luciferase reporter system, the relative luciferase activity of C6 cells transfected with pGL3-AQP4-3UTR and phRL-TK vectors was hindered after co-culture with miR383-RVG exosomes purified from HEK 293 cells transfected with both miR-383-5p mimics shRNA plasmid and the pRVG-Lamp2b plasmid (Figure 5D).

To investigate the potential of RVG exosomes specially delivering miR-383-5p to hippocampus region, unmodified exosomes (vehicle exosomes) or miR383-RVG exosomes were intravenously (IV) injected into SD rats, respectively. Rats were sacrificed 24 h after IV administration for miR-383-5p or AQP4 detection. RT-qPCR analysis revealed miR-383-5p concentration was predominantly higher in the hippocampus of rats with miR383-RVG exosomes IV injection, indicating that RVG exosome could pass through the blood–brain barrier and efficiently delivered miR-383-5p to the hippocampus, compared with unmodified exosome (Figure 5E). Similarly, red fluorescence of miR383-RVG exosomes with CellMask dye was predominantly detectable in hippocampus sections taken by confocal microscopy. However, little fluorescence could be observed in the unmodified-exosome rat hippocampus (Supplementary Figure S3). Moreover, RT-qPCR and western blot analysis showed miR-383-5p upregulation in rat hippocampus resulted in a reduction of AQP4 mRNA and protein expression (Figures 5F–H). Thus, targeted delivery of miR-383-5p to the hippocampus by constructed RVG exosomes could inhibit AQP4 expression.

## Discussion

Astronaut exposure to the microgravity environment caused a variety of adaptive changes in the physiological systems and resulted in a series of space adaptation syndromes. The influences of microgravity on the body were very complex and comprehensive, involving various tissues and organs (Mulavara et al., 2018). For example, microgravity could cause the disappearance of hydrostatic pressure, leading to the redistribution of blood and other bodily fluids along the axis in the cranial direction and ultimately resulting in cognitive decline (Newberg, 1994; Messerotti Benvenuti et al., 2011). Thus, space adaptation syndrome became an important obstacle to long-term deep space exploration. Under microgravity, the redistribution of bodily fluids in the direction of head induces a whole cascade of secondary adaptation mechanisms. Because the effects of microgravity on human body were a dynamical, multi-stage, and synergetic change of multiple organs, it required to use systematic thinking to investigate its development mechanism (Garrett-Bakelman et al., 2019). Recently, “Omics” had become an important method of systematic biomedical research. It could provide large-scale, high-throughput information to reflect the physiological and pathological changes of the body at multiple levels (transcription, post-transcriptional modification, etc.) focusing on multiple targets (mRNA, miRNA, protein and metabolic product, etc.). Conjoint analysis of the mass omics data to explore the complex mechanisms of adaptation syndrome can provide clues to study space medical problems (Nichols et al., 2006; Capulli et al., 2009; Chakraborty et al., 2014; Grimm et al., 2014; Rea et al., 2016; Mao et al., 2018).

Among of these “Omics” methods, metabolomics or serum miRNA microarrays could monitor the influences of microgravity on the body at the small molecular levels and contribute to understanding the underlying molecular mechanisms of microgravity-induced disorders (Chen et al., 2016; Beheshti et al., 2018). In particular, multi-omics analysis, for example, combined analysis of metabolome and transcriptome in biofluids, offered new methods for discovering biomarkers and molecular pathways underlying pathophysiological changes (Mastrokolias et al., 2016). In the present study, we first profiled and integrated the serum metabolome and miRNAome from rats undergoing simulated microgravity using hindlimb unloading method, aiming to probe the composition of serum metabolites and circulating microRNAs. Thus, using the UPLC-MS method, we first analyzed serum samples of HU-28 rats to capture the systemic metabolic variation under SMG. The results indicated that a total of 135 metabolites, a series of energy metabolisms, and nervous system–related metabolic pathways were affected by SMG and contributed to significantly different metabolic profiles between HU and control groups (Supplementary Table S2 and Figure 1). Next, we investigated the changes of circulating miRNAome on the effect of SMG. It has been shown that SMG markedly alters circulating miRNAs in the HU model, as there were 23 different detected miRNAs in miRNA microarrays (Figure 2A). RT-qPCR further validated 13 miRNAs were upregulated from microarrays in the rat serum under SMG (Figure 2B). We also found that the pathways of miRNA target genes were mainly involved in multiple neurological related pathways (Table 2) and the tissue expressions of target genes were mainly related to brain and hippocampus (Table 3) through the data mining by bioinformatics techniques. It was reported that circulating miRNAs could not only work as disease biomarkers but also as a class of hormone to mediate metabolism in body fluids, as so-called miRormone (Cortez et al., 2011; Bayraktar et al., 2017). Thus, we subsequently investigated the correlation of the circulating miRNAome and serum metabolomes in the HU model to narrow the scope of validation. As a result, we found that the top shared pathways (Sudden Infant Death syndrome Susceptibility Pathways, G Protein Signaling Pathways, Dopaminergic synapse, Sphingosine 1-phosphate pathway, Vasopressin-regulated water reabsorption, etc.) were mainly related to nervous system function (Table 4). Finally, only miR-383-5p from 13 observed global circulating miRNAs and AQP4 as one of its predicted target genes were validated in hippocampus of HU rat at different times and found to be significantly influenced by HU (Figure 3). Therefore, it is suggested that miRNAs such as miR-383-5p may be involved in simulated microgravity affecting the nervous system, especially in the hippocampus, and AQP4 may be the target gene of miR-383-5p.

Aquaporin 4 belonged to the aquaporin water channel family (Jung et al., 1994; Beitz and Schultz, 1999) and mainly abundant in brain astrocytes, capillary endothelial cells, and ependymal cells, and was prominently expressed in brain area including the supraoptic nucleus, hippocampal dentate gyrus, and cerebellum (Yool, 2007; Vandebroek and Yasui, 2020). AQP4 played an important role in regulating water balance in brain tissue, contributed to the reabsorption of cerebrospinal fluid and osmotic equilibrium, and closely related to the occurrence of cerebral edema (Papadopoulos et al., 2002; Gandham et al., 2017; Jullienne et al., 2018; Hindeya Gebreyesus and Gebrehiwot Gebremichael, 2020). Previous results showed that the water permeability of astrocytes was significantly decreased after the knockout of AQP4 and reduced the occurrence of cerebral edema (Manley et al., 2000; Papadopoulos and Verkman, 2005; Fu et al., 2007). Besides its vital function in water transport across the blood–brain barrier (Bellone et al., 2016), AQP4 in astrocytes played an unanticipated role in the cognitive function of the hippocampus (Gao et al., 2019). Long-term potentiation and long-term depression were impaired in AQP4 knock-out (KO) mice (Skucas et al., 2011). The impairment of long-term potentiation in AQP4 knock-out mice might be mediated by the reduction of glutamate transporter-1 expression, which suggested that AQP4 can affect memory formation (Yang et al., 2013). Animal behavior experiments also showed the effect of AQP4 on cognitive function. By Morris water maze task and contextual fear conditioning, it was found that the spatial learning and memory ability of mice were significantly decreased, the fear memory was weakened, and the cognitive function was impaired after AQP4 knock out (Liu et al., 2012; Fan et al., 2013; Yang et al., 2013; Zhang et al., 2013).

Microgravity or SMG had an important effect on the blood and body fluid circulation. During exposure to microgravity, gravitational hydrostatic pressure was disappearing so that fluid in the lower extremities immediately shifted to the upper body, especially head tissue (Hargens and Richardson, 2009). The fluid shifts into the head could cause facial edema, orbital swelling, eye thickening, and other symptoms. This phenomenon was termed “puffy face syndrome” (Ewald et al., 2000). It resulted in cerebral excessive hyperemia, which affected the blood supply to the brain and finally caused concentration problems, motion sickness, vision degradation, and other potentially dangerous encephalopathies. The mechanism of microgravity-induced cognitive impairment in the hippocampus is still unclear so far. Jeyaseelan et al. (2008) investigated the involvement of miRNA regulation in brain pathogenesis associated with middle cerebral artery occlusion and found that the expression of both miR-383-5p and AQP4 changed oppositely, according to the data of miRNA and DNA microarray, but they did not further validate this result. Immunoreactivity of AQP4 staining was significantly increased in the brain of hindlimb unloading and irradiation combination group after 9 months (Bellone et al., 2016) or 30 days after low-dose radiation + HU (Mao et al., 2017), and they concluded that combined microgravity and low-dose gamma radiation may lead to blood–brain barrier dysfunction. In the present study, we found a decreased expression of AQP4 during the HU condition, but we did not explore the changes of AQP4 during its recovery. Moreover, the dual-luciferase reporter system monitoring the transcriptional level of the AQP4 affected by the miR-383-5p action revealed that AQP4 was a direct target of miR-383-5p in rat C6 glioma cell *in vitro* (Figure 4). AQP4 not only played a vital role in water transport across the blood–brain barrier but also played an unanticipated role in cognitive function of the hippocampus. Thus, we speculated that microgravity could regulate the expression of the AQP4 by affecting the miR-383-5p and was the potential mechanism of microgravity-induced cognitive impairment in the hippocampus, also related to cerebral excessive hyperemia. As we all know, miRNA was an important method for target gene silencing and became a potentially useful tool for gene therapy recently. However, the traditional methods for microRNA delivery were hard to specifically direct at the target tissues and could induce immune and toxicity responses when used *in vivo* (Chen and Zhaori, 2011; Pecot et al., 2011). Thus, it became the major barrier to the successful clinical application of miRNA. Because nano-sized exosomes secreted from engineered cells may realize accurately and safely delivering small RNA at a specific cell or organizational environment, it compensated for the shortcomings of conventional microRNA delivery carriers and potentially opens up new avenues for future miRNA-based targeted therapy (El Andaloussi et al., 2013; Zhou et al., 2016). Therefore, we used RVG-modified exosomes to verify that delivery of miR-383-5p could inhibit the expression of AQP4 not only in rat C6 glioma cells *in vitro* but also in the hippocampus *in vivo* (Figure 5).

In summary, for the first time we integrated the serum metabolome and miRNAome from rats undergoing hindlimb suspension to probe the composition of serum metabolites and circulating miRNA. Through multi-omics analysis, we found microgravity could promote a miR-383-5p level in the circulation, which could inhibit the expression of AQP4 in hippocampus and be relative to the potential mechanism of microgravity-induced cognitive impairment. Our results also suggested there was a systemic response and regulation mechanism during spaceflight microgravity. The detailed effects and mechanisms of microgravity on miR-383-5p in serum and hippocampus should be further investigated.

## Data Availability Statement

The microarray data has been deposited into the Gene Expression Omnibus (accession: GSE153681).

## Ethics Statement

The animal study was reviewed and approved by the Animal Care Committee of China Astronaut Research and Training Center.

## Author Contributions

ZD, YuL, and YiL designed the study and reviewed the manuscript. HZ, XL, BD, and HN performed the experiments of serum metabolome and evaluated the data. HW, JC, KL, CY, ZG, ZX, and FW performed the experiments of circulating miRNAome and evaluated the data. HZ, HW, and JC prepared the figures. HZ and ZD wrote the manuscript. All authors commented on the manuscript.

## Conflict of Interest

The authors declare that the research was conducted in the absence of any commercial or financial relationships that could be construed as a potential conflict of interest.

## Footnotes

1 https://xcmsonline.scripps.edu/

2 http://www.hmdb.ca/

3 http://www.kegg.com/

4 http://impala.molgen.mpg.de/

5 https://david.ncifcrf.gov/

6 http://impala.molgen.mpg.de

## Abbreviations

AQP4: water channel aquaporin-4
BDNF: brain-derived neurotrophic factor
CACNA2D1: voltage-dependent calcium channel subunit alpha-2/delta-1
ESI: electrospray ionization
HEK 293: human embryonic kidney 293 cell line
HU: hindlimb unloading
IMPaLA: Integrated Molecular Pathway-Level Analysis
IV: intravenous.
KCNH8: potassium voltage-gated channel subfamily H member 8
KEGG: Kyoto Encyclopedia of Genes and Genomes
MAP2: microtubule-associated protein 2
MicroCT: Micro-computed tomography
miRNA: microRNA
NTA: nanoparticle tracking analysis
O2PLS-DA: orthogonal partial least squares–discriminant analysis
PCA: principal component analysis
RT-qPCR: reverse transcription-quantitative polymerase chain reaction
RVG: rabies virus glycoprotein
SD: Sprague-Dawley
SLC1A2: excitatory amino acid transporter 2
SMG: simulated microgravity
TEM: transmission electron microscope
UPLC-MS: ultra-performance liquid chromatography–mass spectrometry
UTR: untranslated regions.
