# Supplementary material for: Serum Metabolomics Associating With Circulating MicroRNA Profiles Reveal the Role of miR-383-5p in Rat Hippocampus Under Simulated Microgravity
Source: Front Physiol. 2020 Aug 18;11:939. doi: 10.3389/fphys.2020.00939 (PMC7461998; doi:10.3389/fphys.2020.00939)
Supplement: Supplementary file 1 [file Table_1.DOCX]

#### Supplementary Materials

**Supplementary Figure:**

**
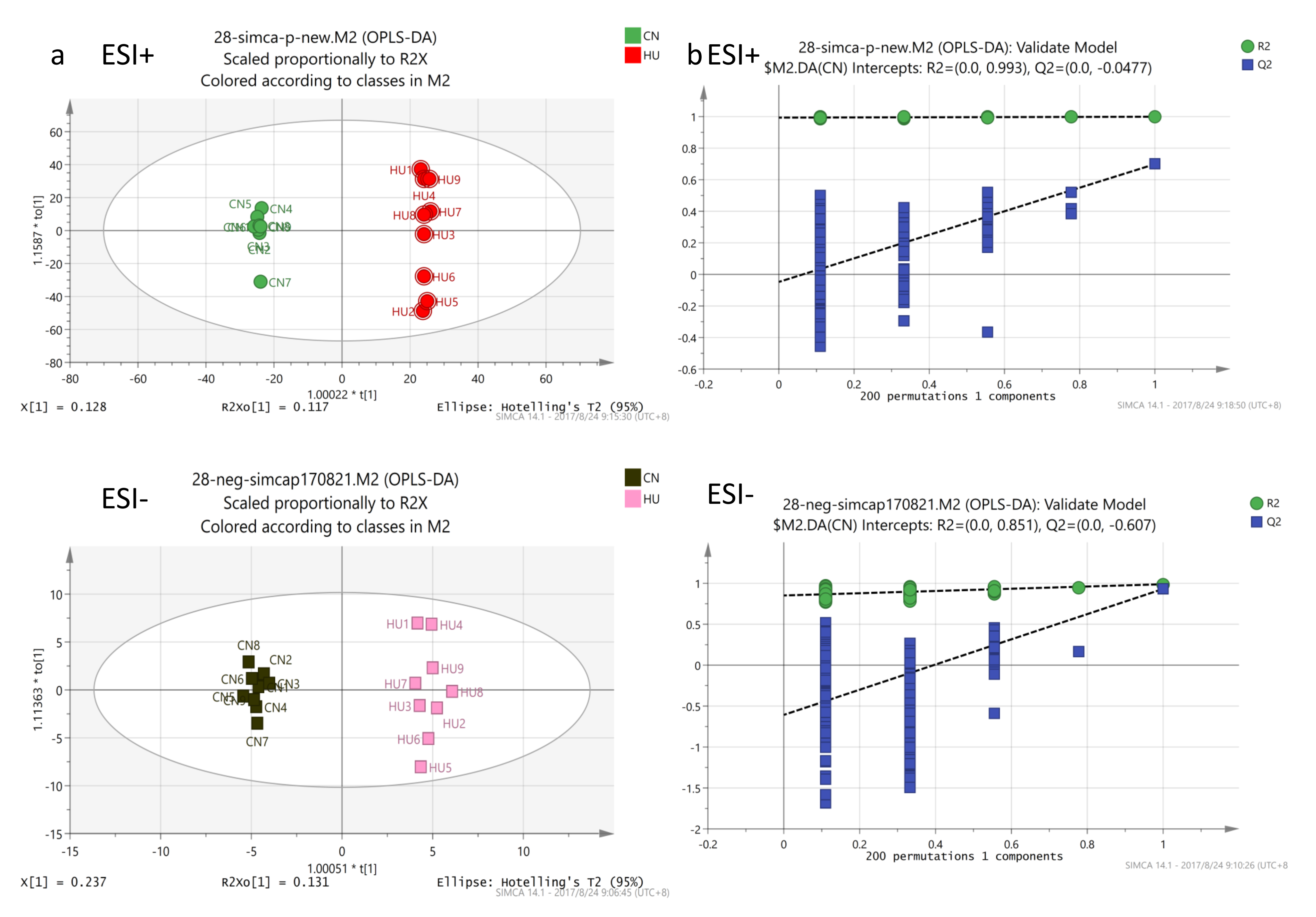
**

**Supplementary Figure S1. OPLS-DA reveals different metabolic profiles between control and HU rats** a. OPLS-DA plot of scores separating HU-28 (red in ESI+ and pink in ESI-) and control (green in ESI+ and black in ESI-) serum samples. b. R2, variation orthogonal (uncorrelated) to the response, and Q2, variation correlated with the response. The vertical axis shows the R2Y and Q2Y values of each model, and the horizontal axis represents the correlation coefficient between the permuted Y-vectors and the original Y-vector. This plot displays the correlation coefficient between the original Y and the permuted Y versus cumulative R2 and Q2.


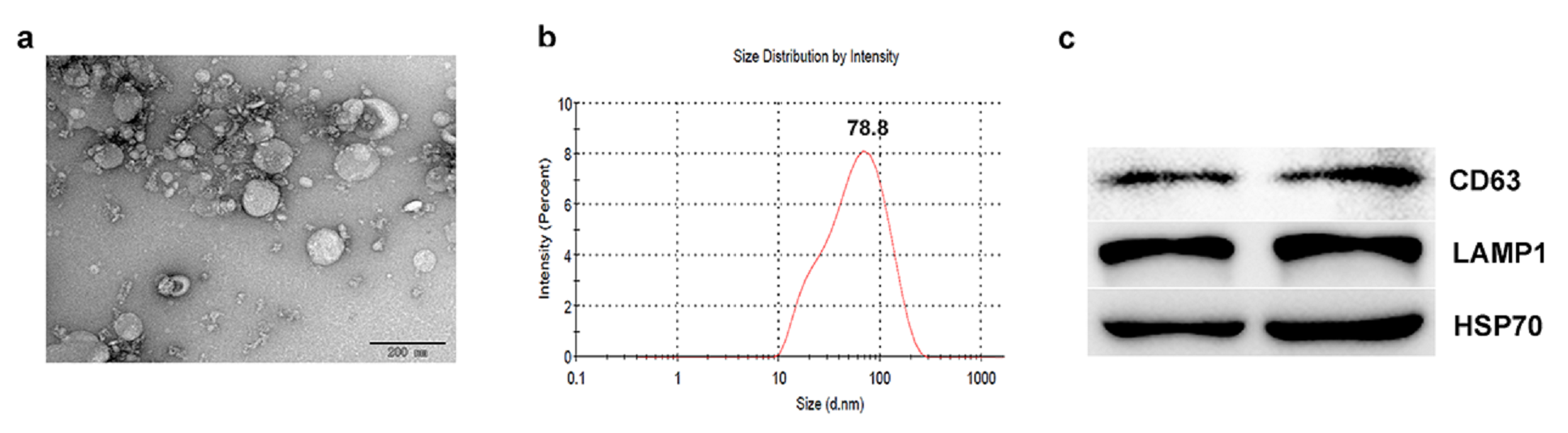


**Supplementary Figure S2. Characterizations of exosomes.** A. Representative TEM image of modified exosomes derived from HEK 293 cells. Scale bar =200 nm. B. NTA profile of modified exosomes from HEK 293 cells Size distributions of exosomes based on NTA. C. Western Blot stained for CD63, Lamp1 and HSP70 on HEK 293-derived RVG-exosomes, the samples derive from the same experiment and the blots were processed in parallel.


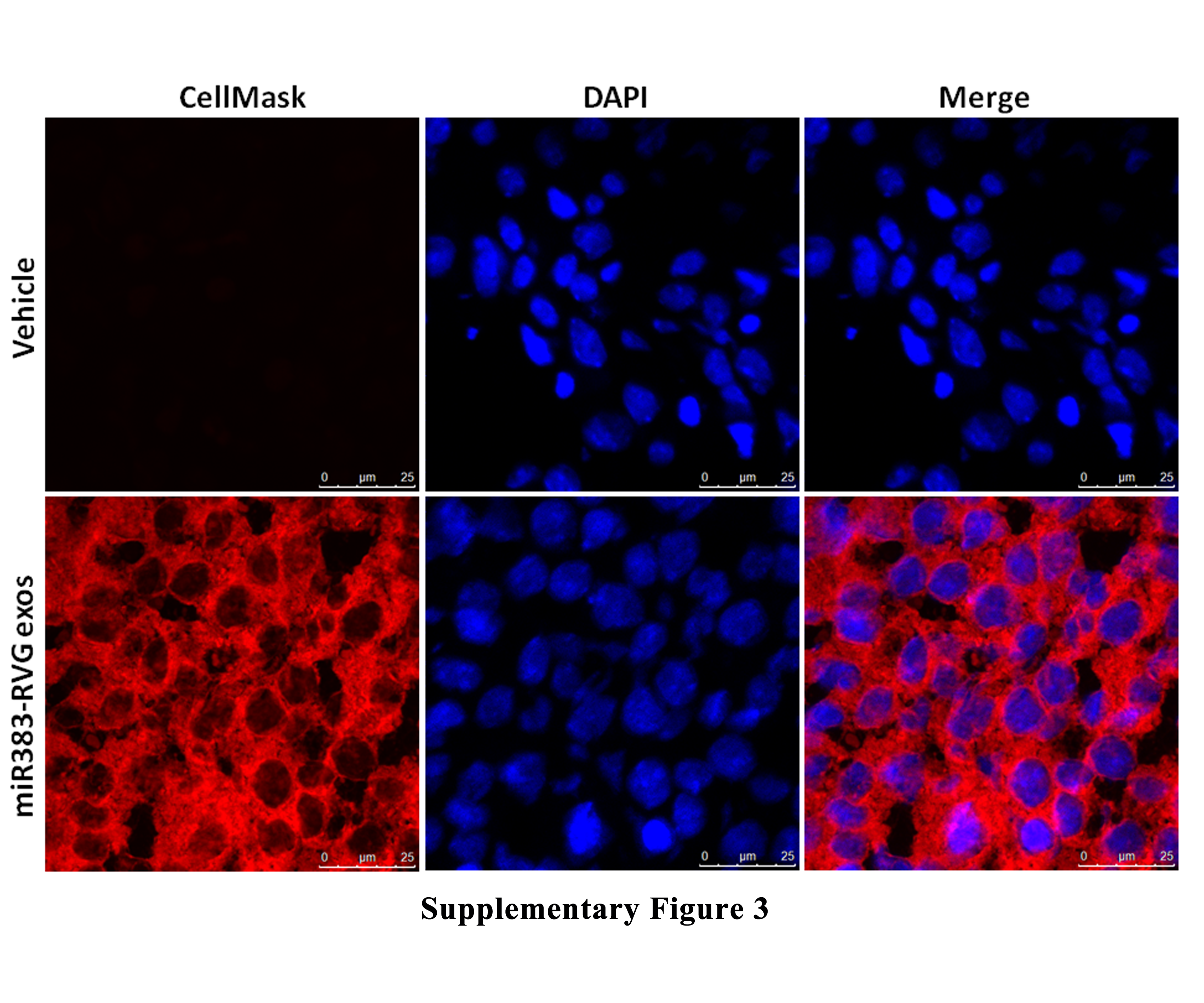


**Supplementary Figure S3. Visualization of miR383-RVG exosomes (labeled with CellMask) in hippocampus by confocal microscopy.** hippocampus samples were collected from rat 24h after iv-administration of saline(above) or miR383-RVG exosomes(below).

**Supplementary tables**

**Table S1 Serum Metabolomics Associating with Circulating MicroRNA Profiles Reveal the Role of miR-383-5p in Rat Hippocampi under Simulated Microgravity**

| Gene | Primer Sequence |
| --- | --- |
| MAP2 | F: CGCAGCGCCAATGGATTTC |
|  | R: TGAACTATCCTTGCAGACACTT |
| AQP4 | F:CATGGAAACCTCACTGCTGGC |
|  | R: TCAGTCCGTTTGGAATCACAG |
| KCNH8 | F: CGACGGAACACATAGCAACTTC |
|  | R: AAGCCATCTGAACAGTAGACT |
| SLC1A2 | F: TGTCCACGACCATCATTGCCG |
|  | R: TTCTTGAGTTTGGGATTCCCA |
| CACNA2D1 | F: ATGCAGCGGTCCATATTCCCA |
|  | R:GCCACAGCAATGTAGGGTCTTCA |
| BDNF | F: TCACAGCGGCAGATAAAAAGA |
|  | R: TACACTTGGTCTCGTAGAAATAT |
| Actin | F: CCGTAAAGACCTCTATGCC |
|  | R: GACTCATCGTACTCCTGCT |
| AQP4-3UTR | F: CGCAGCGCCAATGGATTTC |
|  | R: TGAACTATCCTTGCAGACACTT |
| Lamp2b-5 | F: ATCCGCTAGCGGTCGCCACCATGTGCCTCTCTCCGGTT |
|  | R: GTCACTCGAGCATAAAGGCAAGTACCCTTTGAA |
| Lamp2b-3 | F:GTCACTCGAGGTCACATCCGGAGGTGCAGAATGGGAGATGAATTTCA |
|  | R: ATCCGGATCCTTAGTGTTACAGAGTCTGATATCC |

**Table S2 135 differently metabolites**

| **M/Z** | **VIP** | **FC** | **P Value** | **RT** | **KEGG ID** | **UP/DOWN** |
| --- | --- | --- | --- | --- | --- | --- |
| 115.0379 | 1.32342 | 1.37682 | 0.034519 | 3.9956 | C00430 | UP |
| 171.1 | 1.57082 | 1.453313 | 0.00374 | 4.187558 | C08261 | UP |
| 176.1216 | 1.25801 | 1.312446 | 0.036693 | 1.530667 | C00062 | UP |
| 190.1137 | 1.43605 | 1.422262 | 0.010197 | 4.18765 | C08261 | UP |
| 211.0924 | 1.57135 | 1.440079 | 0.0035 | 4.18765 | C08261 | UP |
| 283.0487 | 1.35161 | 1.278164 | 0.014385 | 4.189958 | C00120 | UP |
| 284.0547 | 1.60727 | 1.373108 | 0.002616 | 4.18765 | C00357 | UP |
| 300.2879 | 1.53902 | 1.550891 | 0.012257 | 6.732517 | C00319 | UP |
| 300.2879 | 1.53902 | 1.550891 | 0.012257 | 6.732517 | C00319 | UP |
| 311.1638 | 1.27396 | 1.158585 | 0.029128 | 10.07762 | C05141 | UP |
| 317.205 | 1.6672 | 2.127396 | 0.005665 | 3.475808 | LMFA03110076 | UP |
| 347.2208 | 1.71197 | 1.447241 | 0.000442 | 4.987867 | C02140 | UP |
| 348.2253 | 1.62707 | 1.440682 | 0.001096 | 4.987883 | C02140 | UP |
| 369.203 | 1.50047 | 1.445175 | 0.007563 | 4.971883 | C02140 | UP |
| 370.2083 | 1.32484 | 1.434238 | 0.027583 | 4.971883 | CE5844 | UP |
| 372.3097 | 1.36161 | 1.605934 | 0.027987 | 6.860542 | ttdcrn | UP |
| 373.3112 | 1.58085 | 1.861676 | 0.006852 | 6.86055 | ttdcrn | UP |
| 385.3444 | 1.24571 | 1.965795 | 0.044948 | 12.53338 | C00599 | UP |
| 395.2764 | 1.51071 | 1.236162 | 0.033801 | 7.484833 | CE5707 | UP |
| 396.1925 | 1.41879 | 2.385177 | 0.034767 | 3.360375 | C16547 | UP |
| 397.2767 | 1.5676 | 1.232216 | 0.032107 | 4.41165 | CE4878 | UP |
| 398.3229 | 1.34573 | 1.854068 | 0.031582 | 7.16475 | hdcecrn | UP |
| 399.3283 | 1.35909 | 1.8532 | 0.030069 | 7.191183 | C01673 | UP |
| 423.2173 | 1.46863 | 1.652878 | 0.026298 | 3.547358 | C05952 | UP |
| 425.2539 | 1.69213 | 2.840103 | 0.004453 | 3.467283 | CE5708 | UP |
| 428.3732 | 1.30857 | 1.566492 | 0.034868 | 9.515525 | stcrn | UP |
| 440.2099 | 1.49491 | 2.273629 | 0.014763 | 3.16325 | CE3481 | UP |
| 443.1639 | 1.77739 | 1.471624 | 0.000801 | 4.18765 | C00440 | UP |
| 519.245 | 1.53649 | 1.588232 | 0.012465 | 3.771367 | C05951 | UP |
| 625.2839 | 1.40856 | 1.930712 | 0.036996 | 6.989067 | C03428 | UP |
| 651.7947 | 1.52494 | 1.628254 | 0.017938 | 3.547425 | C02465 | UP |
| 667.2946 | 1.39456 | 1.83329 | 0.025265 | 5.772217 | C00461 | UP |
| 710.1448 | 1.43018 | 1.374694 | 0.029269 | 4.60735 | C00882 | UP |
| 783.2898 | 1.52183 | 1.867489 | 0.018374 | 3.819467 | C03374 | UP |
| 854.367 | 1.66603 | 2.437854 | 0.006311 | 3.59025 | C15670 | UP |
| 874.2021 | 2.11307 | 1.345628 | 0.00012 | 4.603808 | C05276 | UP |
| 887.6804 | 2.12726 | 1.382991 | 1.41E-05 | 4.603808 | CE5017 | UP |
| 888.1812 | 1.88542 | 1.281663 | 0.000113 | 4.603767 | C05270 | UP |
| 958.2901 | 1.78996 | 2.998033 | 0.003482 | 3.787417 | C05273 | UP |
| 960.3165 | 1.43142 | 1.200384 | 0.00918 | 1.610717 | C02593 | UP |
| 961.2885 | 1.68247 | 3.200218 | 0.006399 | 3.74235 | C02593 | UP |
| 1016.28 | 1.53578 | 1.225658 | 0.004253 | 1.610708 | C02593 | UP |
| 896.3983 | 1.7066 | 6.109098 | 0.000159 | 3.515467 | CE5818 | UP |
| 698.29 | 1.65816 | 6.680064 | 0.000443 | 6.940583 | CE5531 | UP |
| 629.2609 | 1.65177 | 5.887824 | 0.00013 | 3.499567 | C00486 | UP |
| 602.2594 | 1.63439 | 5.088038 | 0.000108 | 3.5155 | CE7086 | UP |
| 399.1778 | 1.55917 | 4.136125 | 3.07E-05 | 4.155667 | C00735 | UP |
| 552.2641 | 1.54614 | 7.129895 | 0.00094 | 3.4354 | clpndcrn | UP |
| 994.4315 | 1.53594 | 4.87768 | 0.00014 | 3.547433 | CE5788 | UP |
| 413.1491 | 1.53342 | 2.733521 | 8.68E-05 | 4.1555 | C04518 | UP |
| 299.0816 | 1.47261 | 3.279529 | 5.38E-06 | 4.764033 | C04148 | UP |
| 446.1924 | 1.40939 | 8.049989 | 0.001012 | 3.675483 | C11133 | UP |
| 419.1642 | 1.38258 | 2.653113 | 5.20E-05 | 4.1555 | C14772 | UP |
| 279.056 | 1.37343 | 5.503341 | 0.003821 | 2.859267 | C00120 | UP |
| 241.0824 | 1.36397 | 3.872938 | 0.001407 | 2.84315 | C00214 | UP |
| 304.0775 | 1.34806 | 3.667332 | 0.000688 | 2.683083 | CE2065 | UP |
| 756.3273 | 1.2967 | 4.022396 | 0.000387 | 9.148483 | CE5791 | UP |
| 661.2716 | 1.28616 | 3.581043 | 0.000231 | 3.499417 | CE4993 | UP |
| 587.3037 | 1.27455 | 5.637058 | 0.008016 | 3.259267 | C03428 | UP |
| 223.074 | 1.27112 | 2.279951 | 0.000117 | 4.155633 | C00214 | UP |
| 287.0876 | 1.26449 | 3.310487 | 0.002249 | 2.84315 | C00214 | UP |
| 599.2513 | 1.25756 | 2.35576 | 5.93E-05 | 3.5153 | C00486 | UP |
| 342.2428 | 1.24837 | 2.959308 | 0.000723 | 10.70188 | C02075 | UP |
| 631.3056 | 1.23954 | 4.069759 | 0.001231 | 6.940733 | CE5790 | UP |
| 311.962 | 1.215 | 3.287125 | 0.000168 | 4.139633 | C07643 | UP |
| 257.0333 | 1.19051 | 2.780503 | 0.000249 | 3.835617 | C00140 | UP |
| 209.079 | 1.18707 | 1.63642 | 3.33E-05 | 4.170317 | C00062 | UP |
| 676.2537 | 1.17544 | 3.466698 | 0.002309 | 5.740167 | CE5531 | UP |
| 445.1888 | 1.15408 | 4.111499 | 0.002516 | 3.67535 | C05504 | UP |
| 277.0596 | 1.14235 | 3.076242 | 0.004081 | 2.843167 | C00214 | UP |
| 447.2014 | 1.12448 | 5.989934 | 0.009997 | 3.62745 | C05503 | UP |
| 354.2147 | 1.0971 | 3.141093 | 0.002762 | 9.543883 | CE5072 | UP |
| 643.2927 | 1.09112 | 2.743174 | 0.000501 | 3.49955 | CE4754 | UP |
| 315.1914 | 1.05956 | 2.46585 | 0.001298 | 3.4354 | C06426 | UP |
| 347.0652 | 1.01094 | 2.495278 | 0.000608 | 3.9636 | CE2947 | UP |
| 958.2901 | 294 | 2.998033 | 0.003482 | 3.771367 | C05273 | UP |
| 425.2539 | 331 | 2.840103 | 0.004453 | 3.451267 | CE5708 | UP |
| 854.367 | 376 | 2.437854 | 0.006311 | 3.563383 | C15670 | UP |
| 961.2885 | 379 | 3.200218 | 0.006399 | 3.739367 | C02593 | UP |
| 373.3112 | 391 | 1.861676 | 0.006852 | 6.844517 | ttdcrn | UP |
| 116.0712 | 1.4905 | 1.280106 | 0.01471 | 1.64935 | C00077 | DOWN |
| 118.0871 | 1.41915 | 1.213553 | 0.019957 | 1.626717 | C00183 | DOWN |
| 119.0907 | 1.51793 | 1.238154 | 0.009691 | 1.639633 | C00183 | DOWN |
| 128.0334 | 1.85369 | 1.291833 | 0.001984 | 1.57875 | C00940 | DOWN |
| 130.0689 | 1.46015 | 1.338362 | 0.015292 | 3.227267 | C00906 | DOWN |
| 133.1024 | 1.54468 | 1.285606 | 0.02372 | 2.715117 | C02576 | DOWN |
| 142.0488 | 1.84681 | 1.312015 | 0.000624 | 1.610708 | C00188 | DOWN |
| 156.0771 | 1.75947 | 1.227028 | 0.002897 | 1.498667 | C00135 | DOWN |
| 162.0546 | 1.63819 | 1.731105 | 0.008856 | 2.7792 | C01586 | DOWN |
| 164.0305 | 1.82296 | 1.299296 | 0.000487 | 1.59475 | C03824 | DOWN |
| 173.0435 | 1.73588 | 1.378546 | 0.003492 | 1.649417 | C03063 | DOWN |
| 174.1099 | 1.28078 | 1.460599 | 0.036636 | 3.8286 | CE2152 | DOWN |
| 178.0588 | 1.59312 | 1.503557 | 0.010922 | 1.530225 | C00135 | DOWN |
| 182.0745 | 1.62053 | 1.446191 | 0.00823 | 1.770775 | C00031 | DOWN |
| 182.0768 | 1.67786 | 1.281007 | 0.00494 | 2.506983 | C07480 | DOWN |
| 188.0676 | 2.05799 | 1.464074 | 0.000102 | 3.227267 | C00079 | DOWN |
| 190.073 | 1.85651 | 1.441594 | 0.001542 | 3.227283 | C00579 | DOWN |
| 190.0841 | 1.30348 | 1.3561 | 0.045966 | 4.6678 | C05587 | DOWN |
| 227.076 | 1.92474 | 1.408269 | 0.00069 | 3.227292 | C16365 | DOWN |
| 228.0794 | 2.0886 | 1.475011 | 0.000169 | 3.227292 | C16365 | DOWN |
| 243.0505 | 2.00645 | 1.629476 | 0.000234 | 3.227275 | C00078 | DOWN |
| 247.1065 | 1.74476 | 2.627833 | 0.003401 | 4.0436 | C04148 | DOWN |
| 269.2262 | 2.20231 | 2.284042 | 1.49E-05 | 10.8042 | C00473 | DOWN |
| 279.1932 | 1.5424 | 1.391672 | 0.009097 | 1.626717 | C16659 | DOWN |
| 285.9888 | 1.61995 | 1.15067 | 0.011912 | 1.528583 | C00018 | DOWN |
| 295.2258 | 1.3002 | 1.50113 | 0.031978 | 7.6129 | C04717 | DOWN |
| 313.0349 | 1.63339 | 1.432511 | 0.007683 | 1.498667 | C00575 | DOWN |
| 317.2098 | 1.43605 | 1.637646 | 0.032445 | 4.85985 | C05953 | DOWN |
| 317.2098 | 1.43605 | 1.637646 | 0.032445 | 4.85985 | LMFA03110075 | DOWN |
| 341.1062 | 1.62633 | 1.998599 | 0.011188 | 3.547367 | C05403 | DOWN |
| 344.094 | 1.66846 | 1.345073 | 0.004305 | 1.610725 | C06157 | DOWN |
| 377.268 | 1.33021 | 2.018302 | 0.039999 | 10.50583 | CE5707 | DOWN |
| 382.2712 | 1.49266 | 1.264556 | 0.049542 | 6.94065 | C01120 | DOWN |
| 410.1878 | 2.0082 | 1.820092 | 0.000204 | 3.227275 | C07108 | DOWN |
| 431.315 | 1.45192 | 1.457381 | 0.025888 | 7.596825 | C17337 | DOWN |
| 449.3052 | 1.32164 | 3.473643 | 0.048805 | 5.003892 | CE7074 | DOWN |
| 483.3271 | 2.07823 | 1.488715 | 0.000351 | 7.500733 | C04840 | DOWN |
| 504.306 | 1.90853 | 1.449251 | 0.001871 | 7.500833 | C03640 | DOWN |
| 505.31 | 1.8485 | 1.386402 | 0.003197 | 7.500833 | C05849 | DOWN |
| 536.3715 | 2.27431 | 1.899386 | 7.69E-06 | 9.583492 | HMDB11521 | DOWN |
| 548.3718 | 2.07433 | 2.001425 | 0.000101 | 9.231008 |  | DOWN |
| 568.3399 | 1.63785 | 1.692656 | 0.00933 | 7.564883 |  | DOWN |
| 690.2463 | 1.64282 | 1.375525 | 0.008974 | 7.564808 | m2mn | DOWN |
| 777.6911 | 2.25293 | 2.022822 | 4.51E-06 | 4.875867 | C01829 | DOWN |
| 846.6616 | 1.43547 | 1.415682 | 0.021823 | 5.06785 | C11378 | DOWN |
| 870.2773 | 2.0751 | 2.093836 | 0.000124 | 3.227267 | C00125 | DOWN |
| 1058.327 | 1.54998 | 1.615225 | 0.015222 | 5.0359 | CE4822 | DOWN |
| 176.0257 | 1.82462 | 2.308134 | 0.000321 | 1.6108 | C14090 | DOWN |
| 208.984 | 1.78139 | 6.024534 | 0.000106 | 1.594883 | C00417 | DOWN |
| 228.995 | 1.74895 | 7.133823 | 0.000177 | 1.5948 | C00158 | DOWN |
| 175.0245 | 1.72809 | 5.595987 | 0.000446 | 1.610417 | C00072 | DOWN |
| 582.368 | 1.71879 | 1.919917 | 0.001786 | 9.532217 | C05200 | DOWN |
| 306.0514 | 1.67226 | 5.337595 | 0.000828 | 1.642283 | C00239 | DOWN |
| 226.994 | 1.66008 | 4.025857 | 0.001429 | 1.5948 | C00074 | DOWN |
| 269.2262 | 10 | 2.284042 | 1.49E-05 | 10.78273 | C00473 | DOWN |

**Table S3 Target gens of miR-383-5p**

| Targetscan(all) | miRanda(-0.8) | 263 shared target genes |
| --- | --- | --- |
| ADIPOQ | ACAT2 | CLIC5 |
| CLIC5 | AK4 | DLG3 |
| CNR1 | AQP9 | GLUD1 |
| CPD | BHLHE40 | HNRNPU |
| CSNK2A1 | BTF3 | LIPA |
| DLC1 | CAST | MAP2 |
| DLG1 | CDS1 | NPR3 |
| DLG2 | CHP1 | NRP2 |
| DLG3 | CKLF | PC |
| DLG4 | CLIC5 | RAB11B |
| DSC2 | CNP | SMS |
| EIF3A | CSN2 | Mar 3 |
| ELK1 | DLG3 | SELT |
| FGF2 | DRG1 | GGACT |
| GALNT7 | EBP | ACSL4 |
| GATC | ELF1 | ADCYAP1 |
| GLUD1 | EMB | AK2 |
| GPI | ERP29 | AK4 |
| HES1 | F3 | AKAP1 |
| HK2 | FAAH | AKAP2 |
| HLF | FBP2 | AKAP6 |
| HNRNPU | FLI1 | AKAP7 |
| HTT | GABPB1 | ALCAM |
| INO80 | GAR1 | ANK3 |
| LIPA | GLUD1 | AP3M1 |
| LRP1 | HCRTR2 | AQP11 |
| LRP4 | HNRNPA1 | **AQP4** |
| MAK | HNRNPU | ARCN1 |
| MAP2 | HSD17B7 | ARF1 |
| MED1 | HSPB8 | ARFIP1 |
| MET | ISPD | ARL8B |
| MGA | LCP1 | ATL1 |
| MIP | LGTN | AXIN2 |
| KMT2D | LIPA | BDNF |
| MPP2 | LRRN1 | BICD2 |
| MSL1 | MAG | BMP3 |
| MTF1 | MAP2 | BTLA |
| MYT1 | MGAT2 | CABP7 |
| NLK | MRLC2 | CABS1 |
| NPR3 | NET1 | CACNA1C |
| NRP2 | NOS3 | CACNA2D1 |
| PAN3 | NPR3 | CALB1 |
| PC | NRP2 | CALM1 |
| PRDX6 | ODC1 | CAMLG |
| RAB11B | PC | CAND1 |
| RGMA | RAB11B | CARM1 |
| RS1 | RHOB | CASK |
| SLC23A2 | RNASE9 | CAST |
| SMS | SAT1 | CCDC71 |
| SNX30 | SEC22B | CCKBR |
| SOCS7 | SMS | CCNA2 |
| SPIN1 | STAP1 | CDKN1B |
| SRGAP2 | SULT1C2 | CELF1 |
| STAG1 | TCEA1 | CHCHD4 |
| SYNDIG1 | TNFRSF11B | CIRH1A |
| TCF4 | TPH2 | CLDN1 |
| TTL | 42065 | CNTN4 |
| VTI1A | 42066 | COL2A1 |
| ZC3H18 | ABCB1A | COPS2 |
| 42066 | AKR1C13 | CREB3L2 |
| 42068 | ANDPRO | CREBBP |
| 42070 | BTNL1 | CRELD1 |
| 42069 | CGM4 | CSNK1G3 |
| D4S234E | CLCN4-2 | CTSB |
| HMP19 | CML1 | CXCL13 |
| HSPE1-MOBKL3 | CYP2A2 | CYB5R4 |
| LOC100127983 | CYP2B21 | CYGB |
| LOC100507203 | CYP2C23 | DAGLA |
| LOC100507421 | CYP2D3 | DCLK1 |
| LOC388630 | DMRTC1A | DEK |
| LOC400950 | ES22 | DLL1 |
| LOC728392 | GIMAP9 | DNAJB9 |
| LPPR5 | GIOT1 | DTNB |
| SELT | GLRX1 | DUSP13 |
| A1CF | HSD3B5 | DYNC1LI2 |
| GGACT | IFI47 | EIF2S2 |
| ABCA1 | IL6RA | EIF4B |
| ABCB7 | KEG1 | EIF4E |
| ABCD3 | KLRA2 | EIF5 |
| ABHD2 | LOC257650 | ELAVL2 |
| ABL2 | LOC298139 | EML2 |
| ACADSB | LOC299282 | ENAH |
| ACAN | LOC302576 | ENC1 |
| ACAP2 | LOC302680 | EPN2 |
| ACSL4 | LOC303448 | ERRFI1 |
| ACTG1 | LOC305806 | ESRRG |
| ACVR1 | LOC317471 | FAF2 |
| ACVR1C | LOC361016 | ABHD17B |
| ACVR2A | LOC361776 | FAM118B |
| ACVR2B | LOC497978 | FAR1 |
| ADAM17 | LOC498368 | FBXO11 |
| ADAMTS15 | LOC498400 | FICD |
| ADAMTS18 | LOC499331 | FMR1 |
| ADAMTS5 | LOC500118 | GABRA1 |
| ADAMTSL3 | LOC500445 | GABRA4 |
| ADAR | LPPR4 | GADD45A |
| ADCY2 | LYZ2 | GAP43 |
| ADCY5 | MBL1 | GMFB |
| ADCY6 | MGC112830 | GNAI3 |
| ADCYAP1 | MGC114483 | GNAZ |
| ADD2 | MGC114529 | GNS |
| ADD3 | MGC94199 | GPR180 |
| ADORA1 | MGC94207 | GPR85 |
| AFF1 | MGC95152 | GRIA3 |
| AFF4 | NSG1 | H2AFY |
| AFTPH | OAS1H | HEXIM1 |
| AGAP1 | OBP3 | HIVEP2 |
| AGBL2 | P22K15 | HNMT |
| AGFG1 | PBSN | HNRNPAB |
| AGPAT6 | PEA15A | HSPA5 |
| AHCYL1 | PRL2A1 | HYOU1 |
| AHDC1 | PRL3D1 | IGF1 |
| AK2 | PRL3D4 | IGFBP5 |
| AK4 | PRL7A4 | IGSF11 |
| AKAP1 | PRL8A2 | IL13RA1 |
| AKAP12 | PRL8A3 | IL2 |
| AKAP13 | PRL8A7 | ING3 |
| AKAP2 | PRL8A9 | ITFG3 |
| AKAP6 | PRP15 | ITM2B |
| AKAP7 | PTGES3L1 | JAG1 |
| ALCAM | REG3B | KAT7 |
| AMMECR1 | RGD1303117 | KCND2 |
| AMOT | RGD1303127 | KCNH8 |
| AMOTL1 | RGD1304978 | KCNK10 |
| AMPH | RGD1305314 | KLF4 |
| ANGEL2 | RGD1306410 | LIMK1 |
| ANK1 | RGD1307161 | LMO4 |
| ANK2 | RGD1307315 | LPL |
| ANK3 | RGD1307890 | MAP3K12 |
| ANKFY1 | RGD1308059 | MCFD2 |
| ANKH | RGD1309437 | MDGA2 |
| ANKIB1 | RGD1309534 | MEOX2 |
| ANKRD12 | RGD1309906 | MLLT10 |
| ANKRD28 | RGD1310597 | MMD |
| ANKRD52 | RGD1310794 | MME |
| SOWAHC | RGD1311122 | MOB4 |
| ANO3 | RGD1311307 | MOSPD1 |
| ANO6 | RGD1311899 | MTMR12 |
| ANO8 | RGD1359334 | NASP |
| ANPEP | RGD1359380 | NDEL1 |
| ANTXR2 | RGD69425 | NDFIP1 |
| ANXA11 | RHOX4G | NECAP1 |
| AP1G1 | RNF113A2 | NEDD9 |
| AP1S3 | RT1-A1 | NEGR1 |
| AP2B1 | RT1-A2 | NFIA |
| AP3M1 | RT1-A3 | NFYB |
| AP4E1 | RT1-DMB | NPTN |
| APBA1 | SECTM1A | NPTX1 |
| APLP2 | SELT | NR3C1 |
| APPL1 | SERPINB6B | NABP1 |
| AQP11 | SGP158 | OGT |
| AQP4 | SIAH1A | PABPC1 |
| ARCN1 | SLCO1A1 | PARG |
| ARF1 | SLCO1A4 | PCDHA1 |
| ARFGAP2 | SLCO1A6 | PCDHA10 |
| ARFGEF1 | SLCO6D1 | PCDHA11 |
| ARFIP1 | SLFN8 | PCDHA12 |
| ARHGAP20 | SULT1C2A | PCDHA13 |
| ARHGAP21 | SULT2AL1 | PCDHA2 |
| ARHGAP26 | TEX21 | PCDHA3 |
| ARHGAP36 | TSX | PCDHA4 |
| ARHGAP42 | UST5R | PCDHA5 |
| ARHGAP5 | VCSA1 | PCDHA6 |
| ARHGEF12 | VEGP2 | PCDHA7 |
| ARHGEF17 | VOM2R27 | PCDHA8 |
| ARHGEF18 | VOM2R32 | PCDHAC1 |
| ARHGEF26 | ZFP110 | PCDHAC2 |
| ARHGEF37 | ZFP354A | PCSK1 |
| ARHGEF40 | ZFP367 | PDIA3 |
| ARIH1 | ZFP384 | PGK1 |
| ARIH2 | ZFP385D | PHACTR2 |
| ARL15 | ZFP423 | PHYHIPL |
| ARL3 | ZFP451 | PITPNA |
| ARL6IP1 | ZFP472 | PLAG1 |
| ARL8A | ZFP819 | POU3F4 |
| ARL8B | ZP3R | PPM1B |
| ARMC1 | GGACT | PPP1CB |
| ARMC10 | ABCG2 | PPP1CC |
| ARMC8 | ABHD6 | PPP2R3C |
| ARNT | ACBD3 | PPP6C |
| ARPC2 | ASIC5 | PRKAG2 |
| ARPC4-TTLL3 | ACOT2 | PRPF19 |
| ARPP19 | ACSL1 | PSME3 |
| ARRDC3 | ACSL4 | PSPH |
| ARX | ACTN1 | PTH |
| ASB7 | ADAM2 | PURB |
| ASH1L | ADAMTS1 | RAB21 |
| ASPH | ADCY8 | RAB34 |
| ATF7 | ADCYAP1 | RAD23B |
| ATG14 | ADNP | RALBP1 |
| ATG2B | AGTR1 | RAP1B |
| ATG7 | AGTR2 | RER1 |
| ATL1 | AHR | RERE |
| ATL2 | AIFM1 | REST |
| ATP10A | AIMP1 | RIMS2 |
| ATP10B | AK2 | RNF34 |
| ATP13A3 | AKAP1 | RNF38 |
| ATP1A2 | AKAP2 | RPS6KB1 |
| ATP2A2 | AKAP6 | RSRC2 |
| ATP2B4 | AKAP7 | RTN3 |
| ATP5G3 | ALCAM | S1PR1 |
| ATP6AP2 | ALDH6A1 | SACM1L |
| ATP6V0B | ALG5 | SCAF8 |
| ATP6V1A | ALOX5AP | SCN2A |
| ATP6V1C2 | AMBN | SDCBP |
| ATP7A | AMIGO2 | SERINC3 |
| ATP8A1 | ANK3 | SERP1 |
| ATP8B2 | ANKRD1 | SERPINE1 |
| ATXN1 | ANXA1 | SERTAD2 |
| ATXN1L | ANXA4 | SH2B3 |
| ATXN7 | AP2M1 | SH3BP5 |
| ATXN7L1 | AP3M1 | SH3KBP1 |
| ATXN7L3 | APOH | SIK1 |
| ATXN7L3B | APOOL | SIPA1L3 |
| AXIN2 | AQP1 | SKP1 |
| B4GALT2 | AQP11 | SLC17A8 |
| B4GALT5 | AQP4 | SLC1A2 |
| BACH2 | ARAF | SLC31A1 |
| BAHCC1 | ARCN1 | SLC35A3 |
| BAHD1 | NAA11 | SMAD5 |
| ADGRB3 | ARF1 | SMNDC1 |
| BARHL1 | ARF4 | SNX27 |
| BAZ2A | ARF3 | SP4 |
| BAZ2B | ARF6 | ST8SIA3 |
| BCAR3 | ARFIP1 | STK17B |
| BCDIN3D | ARHGAP17 | STRN3 |
| BCL11A | ARHGEF2 | SULF1 |
| BCL11B | ARHGEF9 | SYAP1 |
| BCL2 | ARL13A | SYCP1 |
| BCL2L11 | ARL8B | SYT1 |
| BCL2L13 | ARMCX3 | TARDBP |
| BCL2L2 | ART3 | TBKBP1 |
| BCL6 | ASAH1 | TGFA |
| BCL7A | ASNS | TGFBR3 |
| BCL7B | ASPA | TMCO1 |
| BCL9L | ASZ1 | TMEM183A |
| BCLAF1 | ATF6B | TMEM33 |
| BCORL1 | ATG9A | TMEM35 |
| BDNF | ATIC | TMEM41B |
| BICD2 | ATL1 | TMEM54 |
| BLMH | ATP12A | TNFAIP1 |
| BMP3 | ATP1B3 | TOB1 |
| BMPR2 | ATP5F1 | TPM3 |
| BNC2 | ATP6V0D1 | TRPC1 |
| BNIPL | ATP6V0D2 | TSPAN3 |
| BRD2 | ATP6V1B2 | TTPA |
| BRPF1 | ATP6V1G2 | UBAC1 |
| BRWD3 | ATXN3 | UBE2B |
| BSDC1 | AXIN2 | UBE2D3 |
| BSN | AZGP1 | UCP3 |
| BTBD11 | AZIN1 | UGGT1 |
| BTBD3 | B3GALNT1 | UMOD |
| BTG2 | B4GALT6 | USP14 |
| BTLA | B4GALT7 | USP48 |
| BTRC | BAG3 | UXS1 |
| BZRAP1 | BAMBI | VPS54 |
| SKIDA1 | BBS2 | VSIG4 |
| C10orf76 | BCAP29 | WWP1 |
| C11orf87 | BCL2L1 | YTHDC1 |
| MYRF | BDNF | YWHAB |
| C12orf4 | BECN1 | YWHAH |
| PIANP | BEST1 | ZBTB44 |
| CCDC184 | BEX1 | ZDHHC7 |
| TMEM260 | BICD2 |  |
| C14orf166 | BLOC1S2 |  |
| C15orf27 | BMP3 |  |
| C16orf52 | BMPR1A |  |
| C16orf72 | BNIP3 |  |
| C17orf85 | BTD |  |
| C18orf25 | BTG1 |  |
| CCDC178 | BTLA |  |
| C1GALT1 | C1QBP |  |
| C1orf115 | CABP7 |  |
| SZRD1 | CACNA1C |  |
| MINOS1 | CACNA2D1 |  |
| C1orf21 | CACNB2 |  |
| SMIM12 | CACYBP |  |
| SDE2 | CADPS |  |
| SUCO | CALB1 |  |
| GID8 | CALCA |  |
| OSER1 | CALCOCO1 |  |
| NOL4L | CALM1 |  |
| C2orf68 | CALML3 |  |
| C2orf71 | CAMLG |  |
| C3orf18 | CAND1 |  |
| EOGT | CAND2 |  |
| C3orf70 | CAPN13 |  |
| C5orf24 | CAPN3 |  |
| SIMC1 | CAPN6 |  |
| C5orf30 | CARM1 |  |
| SMIM15 | CASK |  |
| C5orf64 | CASP2 |  |
| C6orf120 | CASQ2 |  |
| C6orf136 | CBFB |  |
| PXDC1 | CFAP36 |  |
| SOGA3 | CCDC117 |  |
| LINC01600 | CCDC23 |  |
| TMEM242 | CCDC71 |  |
| SAYSD1 | CCDC82 |  |
| TMEM248 | CCKBR |  |
| C7orf60 | CCL20 |  |
| C7orf71 | CCL28 |  |
| TMEM261 | CCL3 |  |
| SPATA6L | CCNA2 |  |
| C9orf69 | CCNDBP1 |  |
| CAB39 | CCNG1 |  |
| CABP7 | CCNH |  |
| CABS1 | CCR1 |  |
| CACHD1 | CDK20 |  |
| CACNA1A | CCT2 |  |
| CACNA1C | CD200 |  |
| CACNA1E | CD24 |  |
| CACNA2D1 | CD244 |  |
| CACNG2 | CD36 |  |
| CADM1 | CD47 |  |
| CADM2 | CD5 |  |
| CADM3 | CD55 |  |
| CALB1 | CD8B |  |
| CALM1 | CDC37L1 |  |
| CALU | CDC5L |  |
| CAMK2A | CDCA3 |  |
| CAMK2B | CDCA4 |  |
| CAMK2G | CDH17 |  |
| CAMK2N2 | CDKN1B |  |
| CAMKK1 | CDKN2AIP |  |
| CAMKV | CDKN2B |  |
| CAMLG | CDKN2C |  |
| CAMSAP1 | CDO1 |  |
| CAND1 | CEACAM1 |  |
| CANX | CEACAM3 |  |
| CAPZA2 | CEBPB |  |
| CARM1 | CELSR3 |  |
| CASK | CENPC |  |
| CASP14 | CEP55 |  |
| CASR | CEPT1 |  |
| CAST | CFH |  |
| CASZ1 | CHCHD4 |  |
| CBL | CHEK1 |  |
| CBLL1 | CHFR |  |
| CBX5 | CHKA |  |
| CBX6 | CHM |  |
| CC2D1B | CHMP5 |  |
| MTCL1 | CHRM3 |  |
| CCDC50 | CHRNA3 |  |
| COA3 | CHRNA5 |  |
| CCDC6 | CHRNG |  |
| CCDC71 | CIRBP |  |
| CCDC85A | CIRH1A |  |
| CCDC85C | CIT |  |
| CCKBR | CLASP2 |  |
| CCNA2 | CLCA2 |  |
| CCND1 | CLCN3 |  |
| CCND2 | CLDN1 |  |
| CCNF | CLDN11 |  |
| CCNI2 | CLDN8 |  |
| CCNJ | CLDND1 |  |
| CCNJL | CLEC14A |  |
| CCNT2 | CABS1 |  |
| CCNY | CLPX |  |
| CCNYL1 | CLU |  |
| CCT3 | CLUAP1 |  |
| CD34 | CMAS |  |
| CDC25B | CMBL |  |
| CDC27 | CMPK1 |  |
| CDC42 | CNBP |  |
| CDC73 | CNGA2 |  |
| CDH11 | CNKSR2 |  |
| CDH2 | CNOT4 |  |
| CDH23 | CNOT8 |  |
| CDH5 | CNTN3 |  |
| CDH6 | CNTN4 |  |
| CDK13 | COL1A2 |  |
| CDK14 | COL2A1 |  |
| CDK19 | COL4A4 |  |
| CDK5R1 | COMMD10 |  |
| CDK6 | COPS2 |  |
| CDKL2 | COQ10B |  |
| CDKN1B | CORO6 |  |
| CDON | COX8A |  |
| CDR2L | CPOX |  |
| CDV3 | CPT1A |  |
| CECR6 | CR1L |  |
| CELF1 | CRCP |  |
| CELF2 | CREB3L2 |  |
| CELF3 | CREBBP |  |
| CELSR2 | CRELD1 |  |
| CENPBD1 | CRH |  |
| CGN | CRISP1 |  |
| CHCHD4 | CRISP2 |  |
| CHD7 | CDK12 |  |
| CHD9 | CRLS1 |  |
| CHERP | CRNKL1 |  |
| CHIC1 | CRY1 |  |
| CHMP4B | CRYZ |  |
| CHRM1 | YBX3 |  |
| CHST11 | CSDE1 |  |
| CHST2 | CSNK1G3 |  |
| CHSY1 | CSRP1 |  |
| CHTF8 | CSTF1 |  |
| CIRH1A | CTHRC1 |  |
| CKS2 | CTLA4 |  |
| CLCN5 | CTSB |  |
| CLDN1 | CTSE |  |
| CLDN18 | CELF1 |  |
| CLDN22 | CWC15 |  |
| CLEC16A | CXCL1 |  |
| CLK4 | CXCL10 |  |
| CLOCK | CXCL11 |  |
| CLTC | CXCL13 |  |
| CLYBL | CXCR4 |  |
| CMPK2 | CXXC5 |  |
| CNN1 | CYB5R4 |  |
| CNNM3 | CYGB |  |
| CNOT6 | CYP1A1 |  |
| CNOT6L | CYP24A1 |  |
| CNST | CYP26A1 |  |
| CNTN4 | CYP7A1 |  |
| CNTNAP1 | CYYR1 |  |
| CNTNAP2 | DAGLA |  |
| COG5 | DBNL |  |
| COL1A1 | DCLK1 |  |
| COL2A1 | DCN |  |
| COL4A1 | DDHD1 |  |
| COL4A3 | DDX46 |  |
| COL5A2 | DEFB1 |  |
| COL5A3 | DEK |  |
| COPS2 | DGAT2 |  |
| CORO2B | DHRS1 |  |
| CPEB2 | DLL1 |  |
| CPEB3 | DLL3 |  |
| CPEB4 | DMAP1 |  |
| CPLX3 | DMGDH |  |
| CPNE9 | DMTF1 |  |
| CPSF6 | DNAI2 |  |
| CPSF7 | DNAJB6 |  |
| CRAMP1L | DNAJB9 |  |
| CREB1 | DNAJC12 |  |
| CREB3L2 | DNAJC2 |  |
| CREB5 | DNASE1L1 |  |
| CREBBP | DNM1L |  |
| CREBZF | DONSON |  |
| CREG2 | DPH2 |  |
| CRELD1 | DPY30 |  |
| CS | DR1 |  |
| CSF1 | DSN1 |  |
| CSNK1A1 | DTNB |  |
| CSNK1D | DTWD1 |  |
| CSNK1G1 | DUOX2 |  |
| CSNK1G2 | DUSP13 |  |
| CSNK1G3 | DUSP2 |  |
| CTC1 | DYNC1I2 |  |
| CTDNEP1 | DYNC1LI2 |  |
| CTGF | DYNC2LI1 |  |
| CTNND1 | DYNLT3 |  |
| CTNND2 | EBAG9 |  |
| CTSB | EFHD2 |  |
| CTTNBP2NL | EFNB1 |  |
| CUL5 | EGLN3 |  |
| CUX1 | EGR1 |  |
| CXCL13 | EI24 |  |
| CXorf23 | EIF2AK3 |  |
| CYB5R4 | EIF2S1 |  |
| CYGB | EIF2S2 |  |
| CYP2B6 | EIF4A2 |  |
| CYTH3 | EIF4B |  |
| DAGLA | EIF4E |  |
| DAZAP2 | EIF4H |  |
| DCAF8 | EIF5 |  |
| DCAKD | ELAVL2 |  |
| DCBLD2 | ELL3 |  |
| DCK | ELOVL6 |  |
| DCLK1 | ADGRL4 |  |
| DCP1A | EML2 |  |
| DCP2 | ENAH |  |
| DCUN1D4 | ENC1 |  |
| DCX | ENO2 |  |
| DDHD2 | EPCAM |  |
| DDX3X | EPDR1 |  |
| DDX3Y | EPHX2 |  |
| DDX42 | EPN2 |  |
| DDX6 | ERGIC2 |  |
| DEDD | ERMN |  |
| DEK | ERMP1 |  |
| DENND1B | ERRFI1 |  |
| DENND4C | ESAM |  |
| DGCR8 | ESM1 |  |
| DGKB | ESRRG |  |
| DGKG | ETS1 |  |
| DHRS13 | F12 |  |
| DHX15 | FABP1 |  |
| DICER1 | FABP4 |  |
| DIP2A | FAF2 |  |
| DIP2B | ABHD17B |  |
| DIP2C | FAM118B |  |
| DLG5 | FAM133B |  |
| DLGAP1 | FAM174A |  |
| DLL1 | FAM26E |  |
| DMD | FAM58BP |  |
| DMPK | FAM71F2 |  |
| DMRT1 | FAR1 |  |
| DMRTA2 | FASLG |  |
| DMXL1 | FAT1 |  |
| DNAJB12 | FAT2 |  |
| DNAJB9 | FBP1 |  |
| DNAJC18 | FBXO11 |  |
| DNAJC27 | FBXO30 |  |
| DNAJC3 | FDX1 |  |
| DNAJC5 | FGF13 |  |
| DNAJC8 | FGF16 |  |
| DNM3 | FGR |  |
| DNMT1 | FICD |  |
| DOCK6 | FIGNL1 |  |
| DOK6 | FILIP1 |  |
| DPP10 | FLVCR2 |  |
| DPP9 | FMR1 |  |
| DPYSL3 | FOS |  |
| DPYSL5 | FOSL1 |  |
| DSEL | FOXG1 |  |
| DTNB | FUNDC1 |  |
| DUSP13 | TIMM10B |  |
| DUSP15 | VWA7 |  |
| DUSP16 | GABRA1 |  |
| DUSP3 | GABRA4 |  |
| DUSP7 | GABRG1 |  |
| DUSP8 | GADD45A |  |
| DYNC1LI2 | GAL |  |
| DYNLL2 | GALNT11 |  |
| DYRK1A | GAP43 |  |
| DYRK1B | GBAS |  |
| DYRK2 | GCLC |  |
| E2F3 | GCLM |  |
| E2F7 | GCNT2 |  |
| EBF1 | GDAP2 |  |
| EDA | GDF10 |  |
| EFEMP1 | GEMIN8 |  |
| MICU3 | GGCX |  |
| EFNA5 | GGPS1 |  |
| EFNB2 | GHRH |  |
| EFR3B | GIPC1 |  |
| EGLN1 | GJA1 |  |
| EGR2 | GJD2 |  |
| AGO1 | GK |  |
| AGO4 | GKAP1 |  |
| EIF2D | GLRA1 |  |
| EIF2S2 | GLRA2 |  |
| EIF4B | GMCL1 |  |
| EIF4E | GMDS |  |
| EIF4E3 | GMFB |  |
| EIF4ENIF1 | GNAI3 |  |
| EIF5 | GNAO1 |  |
| EIF5A2 | GNAZ |  |
| ELAVL2 | GNB1 |  |
| ELF5 | GNG10 |  |
| ELMO1 | GNL3 |  |
| ELMO2 | GNRHR |  |
| EML2 | GNS |  |
| EML6 | GOLPH3L |  |
| EMP1 | GPC2 |  |
| EMX2 | GPC4 |  |
| EN2 | GPR149 |  |
| ENAH | GPR180 |  |
| ENC1 | ADGRG2 |  |
| ENOX2 | GPR83 |  |
| ENSA | GPR85 |  |
| ENTPD7 | GPRASP1 |  |
| EP300 | GRIA3 |  |
| EPAS1 | GRIA4 |  |
| EPB41 | GRID2 |  |
| EPB41L4B | GRIK1 |  |
| EPC2 | PPP1R17 |  |
| EPHA8 | GSPT1 |  |
| EPN2 | CFAP20 |  |
| EPS15 | GUCY1B2 |  |
| ERBB3 | GUCY1B3 |  |
| ERBB4 | GULP1 |  |
| ERC1 | H2AFY |  |
| ERLEC1 | HAL |  |
| ERLIN1 | HAS2 |  |
| ERRFI1 | HCLS1 |  |
| ESCO1 | HEXIM1 |  |
| ESR1 | HHEX |  |
| ESRRA | HIBADH |  |
| ESRRG | HIBCH |  |
| ETNK1 | HIRIP3 |  |
| ETV1 | HIST1H4B |  |
| ETV6 | HIVEP2 |  |
| EXT1 | HMBS |  |
| FAF2 | HMGN1 |  |
| FAM104A | HNMT |  |
| ABHD17B | HNRNPAB |  |
| FAM117A | HNRNPK |  |
| FAM117B | HNRNPH1 |  |
| FAM118B | HOMER3 |  |
| FAM120A | HP1BP3 |  |
| FAM122A | HSD17B11 |  |
| AMER1 | HSPA5 |  |
| FAM124B | HSPD1 |  |
| FAM126B | HTR5A |  |
| FAM131B | SPAM1 |  |
| FAM134C | HYOU1 |  |
| FAM135B | IBSP |  |
| SAXO2 | ID2 |  |
| FAM168B | IFI30 |  |
| EVA1A | IFIT2 |  |
| FAM178A | IGF1 |  |
| FAM184A | IGF2 |  |
| FAM189B | IGFBP1 |  |
| CCSER1 | IGFBP5 |  |
| FAM193B | IGSF11 |  |
| FAM204A | IL10 |  |
| FAM20B | IL13RA1 |  |
| PIEZO1 | IL1RN |  |
| STRIP1 | IL2 |  |
| FAM49A | IL22RA2 |  |
| BRINP2 | ILKAP |  |
| FAM76B | IMPA1 |  |
| FAM81A | IMPG2 |  |
| FAM83F | ING3 |  |
| FAM84B | INO80C |  |
| FAR1 | INPP4B |  |
| FASTKD2 | INSIG1 |  |
| FBN1 | INSIG2 |  |
| FBXL19 | IQCF3 |  |
| FBXL5 | IRF1 |  |
| FBXO10 | IRGM |  |
| FBXO11 | ISG20L2 |  |
| FBXO28 | ISOC1 |  |
| FBXO33 | ITFG1 |  |
| FBXO46 | ITFG3 |  |
| FBXW7 | ITGB6 |  |
| FCHSD1 | ITM2A |  |
| FCHSD2 | ITM2B |  |
| FECH | ITM2C |  |
| FEZF1 | ITPR1 |  |
| FGD4 | JAG1 |  |
| FGF1 | JMJD8 |  |
| FGF12 | JUN |  |
| FGF9 | KATNA1 |  |
| FGFR3 | KCNA2 |  |
| FICD | KCNA3 |  |
| FIGF | KCNA4 |  |
| FLOT2 | KCNC2 |  |
| FMN1 | KCND2 |  |
| FMNL3 | KCNH8 |  |
| FMR1 | KCNJ3 |  |
| FNBP1L | KCNJ4 |  |
| FNDC3A | KCNJ8 |  |
| FNDC3B | KCNK10 |  |
| FOSB | KCNK2 |  |
| FOXA1 | KCNQ3 |  |
| FOXD1 | KCNV1 |  |
| FOXI1 | KIFC2 |  |
| FOXJ2 | KLF10 |  |
| FOXJ3 | KLF4 |  |
| FOXK2 | KLF5 |  |
| FOXN2 | KLHDC2 |  |
| FOXN3 | KLHL10 |  |
| FRK | KLHL24 |  |
| FRMD4A | KLHL7 |  |
| FRMD4B | KPNB1 |  |
| FST | KRCC1 |  |
| FSTL4 | LAMP2 |  |
| FURIN | LAPTM4A |  |
| FUT8 | LARS |  |
| FUT9 | CERS2 |  |
| FXYD3 | LEPROT |  |
| FZD1 | LGR4 |  |
| FZD4 | LHX8 |  |
| G3BP2 | LIMK1 |  |
| GABARAPL1 | LIPC |  |
| GABRA1 | LMO2 |  |
| GABRA4 | LMO4 |  |
| GABRB1 | LNPEP |  |
| GABRG2 | ADGRL3 |  |
| GADD45A | LPL |  |
| GAP43 | LRIT1 |  |
| GAPVD1 | LRRC8C |  |
| GATA2 | LRRFIP1 |  |
| GATA4 | LUC7L |  |
| GATAD2B | LYPD1 |  |
| GDF6 | LYPLA2 |  |
| GGA2 | LZIC |  |
| GGA3 | MAGT1 |  |
| GGT6 | MAK16 |  |
| GIGYF2 | MAL2 |  |
| GIT1 | MAN1A1 |  |
| GKN1 | MANBA |  |
| GLCE | MAP2K1 |  |
| GLI3 | MAP3K12 |  |
| GLRX5 | MAP3K8 |  |
| GMFB | MAPK13 |  |
| GNAI3 | MAPK6 |  |
| GNAZ | MAPK9 |  |
| GNG12 | MAPRE1 |  |
| GNPNAT1 | MATN1 |  |
| GNS | MATR3 |  |
| GOPC | MAX |  |
| GOSR1 | MBD1 |  |
| GPATCH8 | MCFD2 |  |
| GPC6 | MDGA2 |  |
| GPCPD1 | MDH1 |  |
| GPHN | MEOX2 |  |
| GPNMB | MEPE |  |
| ADGRF5 | MESDC2 |  |
| ADGRA2 | METAP2 |  |
| GPR180 | METTL7A |  |
| GPR63 | MID1IP1 |  |
| GPR85 | MLLT10 |  |
| GPRC5B | MLLT11 |  |
| GPSM3 | MMD |  |
| GRHL2 | MME |  |
| GRIA2 | MMP10 |  |
| GRIA3 | MMP3 |  |
| GRID1 | MOB4 |  |
| GRIN3A | MOBP |  |
| GRM5 | MOCS2 |  |
| GRPR | MOSPD1 |  |
| GTDC1 | MRPL17 |  |
| GTF2H1 | MRPL40 |  |
| GTPBP2 | MSMB |  |
| GXYLT1 | MTHFD1 |  |
| GZF1 | MTHFS |  |
| H2AFJ | MTMR12 |  |
| H2AFY | MTRR |  |
| HABP2 | MUS81 |  |
| HBS1L | MX1 |  |
| HCFC1 | MYNN |  |
| HDAC7 | MYOCD |  |
| HDLBP | KAT8 |  |
| HECW2 | KAT7 |  |
| HELZ | NAAA |  |
| HERC2 | NANP |  |
| HEXIM1 | NAP1L2 |  |
| HIATL1 | NARS |  |
| HIC2 | NASP |  |
| HIPK1 | NCDN |  |
| HIPK2 | NDEL1 |  |
| HIVEP1 | NDFIP1 |  |
| HIVEP2 | NDUFS4 |  |
| HIVEP3 | NECAP1 |  |
| HMGA1 | NEDD9 |  |
| HMGB2 | NEFL |  |
| HMGB3 | NEGR1 |  |
| HMGN5 | NEUROD1 |  |
| HNF4G | NFIA |  |
| HNMT | NFYB |  |
| HNRNPA2B1 | NFYC |  |
| HNRNPAB | NGFRAP1 |  |
| HNRNPD | NIF3L1 |  |
| HNRNPH2 | NKAP |  |
| HNRNPUL1 | NKX3-1 |  |
| HNRNPUL2 | NME7 |  |
| HOOK3 | NOL3 |  |
| HOXA7 | NOS1AP |  |
| HOXA9 | NOTCH4 |  |
| HOXB3 | NOX4 |  |
| HOXB8 | NPHS1 |  |
| HOXC10 | NPL |  |
| HOXC5 | NPTN |  |
| HOXC6 | NPTX1 |  |
| HOXC8 | NPY |  |
| HOXD10 | NQO2 |  |
| HPSE2 | NR0B1 |  |
| HS2ST1 | NR3C1 |  |
| HS6ST1 | NR4A1 |  |
| HSPA4L | NR4A3 |  |
| HSPA5 | NT5E |  |
| HSPB7 | NTF4 |  |
| LGALSL | NTM |  |
| HTR4 | NUCKS1 |  |
| HYOU1 | NUDCD2 |  |
| ICMT | NUDT3 |  |
| IFFO2 | NUP93 |  |
| IGDCC4 | NUPL1 |  |
| IGF1 | NUTF2 |  |
| IGF1R | NXPH1 |  |
| IGF2BP1 | NABP1 |  |
| IGF2R | ODF2 |  |
| IGFBP5 | ODF4 |  |
| IGSF11 | OGT |  |
| IHH | OLFML1 |  |
| IKBKE | OLIG1 |  |
| IL13RA1 | OPCML |  |
| IL2 | OPTN |  |
| IL6ST | ORC5 |  |
| IMPAD1 | OS9 |  |
| ING1 | OSBPL2 |  |
| ING3 | OSGEPL1 |  |
| INHBB | OSTF1 |  |
| INO80D | OTC |  |
| INSC | P2RY1 |  |
| INSM1 | P2RY14 |  |
| INTS2 | PABPC1 |  |
| IP6K1 | PADI4 |  |
| IPO5 | PAIP2 |  |
| IRF4 | PANX1 |  |
| ITFG3 | PANX3 |  |
| ITGA1 | PARG |  |
| ITGA11 | PAWR |  |
| ITGA2 | PAX6 |  |
| ITGA5 | KAT2B |  |
| ITGA6 | PCDH7 |  |
| ITGA9 | PCDHA1 |  |
| ITGB8 | PCDHA10 |  |
| ITM2B | PCDHA11 |  |
| ITPK1 | PCDHA12 |  |
| ITSN1 | PCDHA13 |  |
| ITSN2 | PCDHA2 |  |
| JAG1 | PCDHA3 |  |
| JAG2 | PCDHA4 |  |
| JAKMIP2 | PCDHA5 |  |
| JPH1 | PCDHA6 |  |
| JPH3 | PCDHA7 |  |
| AJUBA | PCDHA8 |  |
| KALRN | PCDHAC1 |  |
| KAT7 | PCDHAC2 |  |
| KAZN | PCGF6 |  |
| KCMF1 | PCK1 |  |
| KCNA1 | PCSK1 |  |
| KCNAB1 | PCTP |  |
| KCNC1 | PDCL |  |
| KCND2 | PDE12 |  |
| KCNH1 | PDE5A |  |
| KCNH8 | PDIA3 |  |
| KCNIP2 | PDPN |  |
| KCNIP3 | PENK |  |
| KCNJ10 | PEX3 |  |
| KCNK10 | PFKM |  |
| KCNQ4 | PGAM5 |  |
| KCNQ5 | PGK1 |  |
| KCTD3 | PGRMC1 |  |
| KDM6B | PHACTR2 |  |
| KIAA0040 | PHKG2 |  |
| KIAA0141 | PHYHIPL |  |
| KIAA0232 | PIAS3 |  |
| KIAA0355 | PICALM |  |
| KIAA0408 | PIR |  |
| CLUH | PITPNA |  |
| SOGA1 | PITX2 |  |
| KIAA0907 | PJA2 |  |
| KIAA1033 | PKIB |  |
| KIAA1217 | PLAG1 |  |
| KANSL1 | PLAT |  |
| PALD1 | PLCB4 |  |
| KIAA1324L | PLCL1 |  |
| KIAA1456 | BLOC1S6 |  |
| KIAA1468 | PLSCR1 |  |
| KIAA1549 | PMCH |  |
| KIAA2018 | PNO1 |  |
| KIAA2022 | POLR2G |  |
| KIF1B | POLR3K |  |
| KIF21A | POMT1 |  |
| KIF21B | PON3 |  |
| KIF23 | POT1 |  |
| KIF3B | POU1F1 |  |
| KIF5A | POU3F4 |  |
| KIT | PPAP2B |  |
| KLC2 | PPIF |  |
| KLF12 | PPM1B |  |
| KLF13 | PPP1CB |  |
| KLF3 | PPP1CC |  |
| KLF4 | PPP1R2 |  |
| KLF6 | PPP1R3C |  |
| KLHL18 | PPP1R7 |  |
| KLHL20 | PPP2R3C |  |
| KLHL28 | PPP4C |  |
| KLHL3 | PPP6C |  |
| KPNA1 | PRIM1 |  |
| KPNA3 | PRKAG2 |  |
| KPNA4 | PRKCD |  |
| KRAS | PRKX |  |
| KY | PRPF19 |  |
| LAMA4 | PRPS2 |  |
| LAMP1 | PRRX1 |  |
| LANCL3 | PSENEN |  |
| LARP1 | PSIP1 |  |
| LARP4B | PSMB9 |  |
| LASP1 | PSME3 |  |
| LATS2 | PSPC1 |  |
| LBR | PSPH |  |
| LCOR | PTF1A |  |
| LDLR | PTH |  |
| P3H2 | PTP4A1 |  |
| LHFPL2 | PTPN21 |  |
| LHX2 | PTPN5 |  |
| LIFR | PTPRA |  |
| LIMCH1 | PTPRR |  |
| LIMD2 | PURB |  |
| LIMK1 | PXMP2 |  |
| LIN28A | PXMP4 |  |
| LIN54 | QPRT |  |
| LMBR1L | QRSL1 |  |
| LMO4 | RAB1A |  |
| LMTK2 | RAB11A |  |
| LPAR2 | RAB21 |  |
| LPGAT1 | RAB27A |  |
| LPL | RAB2B |  |
| LRCH1 | RAB34 |  |
| LRCH2 | RAB7A |  |
| LRFN1 | RAB9A |  |
| LRP1B | RABIF |  |
| LRRC10B | RAD21 |  |
| LRRC32 | RAD23B |  |
| LRRC4 | RALBP1 |  |
| LRRC4C | RALYL |  |
| LRRC55 | RAN |  |
| LRRC8B | RANGAP1 |  |
| LRTOMT | RAP1A |  |
| LSAMP | RAP1B |  |
| LTBP1 | RASGRP1 |  |
| LUC7L3 | RBL2 |  |
| LYPD3 | RBM12 |  |
| MAB21L1 | SCAF8 |  |
| MAF | RBM22 |  |
| MAF1 | RBM45 |  |
| MAFB | RBM47 |  |
| MAFG | RBM4B |  |
| MAGI2 | RBMS1 |  |
| MAML1 | RBX1 |  |
| MAP1B | RELA |  |
| MAP3K12 | RER1 |  |
| MAP3K2 | RERE |  |
| MAP3K3 | REST |  |
| MAP3K5 | REXO2 |  |
| MAP3K7 | RFK |  |
| MAP3K9 | TRMT10B |  |
| MAPK1 | RGN |  |
| MAPK7 | RGS2 |  |
| MAPKAPK3 | RGS7 |  |
| MAPKBP1 | RIMS1 |  |
| MARCKS | RIMS2 |  |
| MARK2 | RND3 |  |
| MASP1 | RNF103 |  |
| MBLAC2 | RNF126 |  |
| MBNL1 | RNF146 |  |
| MBOAT2 | RNF181 |  |
| MBTPS2 | 42073 |  |
| MCF2 | RNF34 |  |
| MCFD2 | RNF38 |  |
| MDFIC | RNLS |  |
| MDGA1 | RNPEP |  |
| MDGA2 | ROCK2 |  |
| MDM4 | ROS1 |  |
| MED12L | RPAP2 |  |
| MED13 | RPL8 |  |
| MED14 | POLR1D |  |
| MED26 | RPS14 |  |
| MEF2C | RPS6KB1 |  |
| MEF2D | RRAGA |  |
| MEGF11 | RRAGB |  |
| MEOX2 | RRP8 |  |
| MEX3A | RSAD2 |  |
| MEX3B | RSL24D1 |  |
| MEX3C | RSRC2 |  |
| MFAP3 | RTN3 |  |
| MFSD6 | RUVBL2 |  |
| MGAT4A | S1PR1 |  |
| MIB1 | SACM1L |  |
| MID1 | SAR1A |  |
| MIDN | MSMO1 |  |
| MIER1 | SCG2 |  |
| MIER3 | SCLT1 |  |
| MIPOL1 | SCN2A |  |
| MITF | SCN3B |  |
| MLEC | SCOC |  |
| KMT2A | SDC2 |  |
| MLLT10 | SDC4 |  |
| MLLT3 | SDCBP |  |
| MLLT6 | SDHAF2 |  |
| MMAA | SELE |  |
| MMD | SEMA4F |  |
| MME | SEMG1 |  |
| MMP15 | SERINC3 |  |
| MMP16 | SERP1 |  |
| MMS22L | SERPINA10 |  |
| MNT | SERPINB2 |  |
| MOB1B | SERPIND1 |  |
| MOB3B | SERPINE1 |  |
| MOB4 | SERTAD2 |  |
| MOGS | SET |  |
| MON2 | SFRP4 |  |
| MORC1 | SREK1IP1 |  |
| 42064 | SRSF7 |  |
| MOSPD1 | SFXN1 |  |
| MOSPD2 | SFXN2 |  |
| MPP3 | POMK |  |
| MPPED1 | SGMS1 |  |
| MPPED2 | SH2B3 |  |
| MRAS | SH3BP5 |  |
| MSH6 | SH3GL3 |  |
| MSI2 | SH3GLB1 |  |
| MSL2 | SH3KBP1 |  |
| MTDH | SHOX2 |  |
| MTMR10 | SIL1 |  |
| MTMR12 | SIPA1L3 |  |
| MTMR14 | SIRPA |  |
| MTMR3 | SIRT2 |  |
| MTMR4 | SIRT5 |  |
| MTPN | SKP1 |  |
| MTSS1L | SLC12A2 |  |
| MUM1L1 | SLC14A2 |  |
| MXD4 | SLC16A1 |  |
| MYB | SLC17A8 |  |
| MYBL1 | SLC18A2 |  |
| MYCL | SLC1A1 |  |
| MYH7B | SLC1A2 |  |
| MYO1E | SLC20A1 |  |
| MYO3A | SLC21A4~withdrawn |  |
| MYO6 | SLC22A2 |  |
| MYT1L | SLC22A5 |  |
| N4BP1 | SLC25A1 |  |
| NAA15 | SLC25A19 |  |
| NADK2 | SLC25A27 |  |
| NASP | SLC31A1 |  |
| NAT14 | SLC34A1 |  |
| NAT8L | SLC35A3 |  |
| NAV1 | SLC38A1 |  |
| NAV2 | SLC38A3 |  |
| NCAM1 | SLC4A10 |  |
| NCBP2 | SLC6A15 |  |
| NCKIPSD | SLC7A3 |  |
| NCOA1 | SLC9A3 |  |
| NCOA2 | SLC9A4 |  |
| NCS1 | SLCO1B3 |  |
| NDEL1 | SLCO4A1 |  |
| NDFIP1 | SMAD4 |  |
| NDOR1 | SMAD5 |  |
| NDRG3 | SMAD7 |  |
| NECAB1 | SMC1A |  |
| NECAP1 | SMNDC1 |  |
| NEDD9 | SNAI1 |  |
| NEGR1 | SNCA |  |
| NEIL2 | SIK1 |  |
| NEK1 | SNX10 |  |
| NETO1 | SNX16 |  |
| NEURL1 | SNX27 |  |
| NEURL4 | SNX7 |  |
| NF1 | SORBS2 |  |
| NFAT5 | SP4 |  |
| NFIA | SPATA1 |  |
| NFIB | SPATA6 |  |
| NFIX | SPATS1 |  |
| NFYA | SSB |  |
| NFYB | SSTR1 |  |
| NHS | ST18 |  |
| NIPBL | ST6GALNAC3 |  |
| NKTR | ST8SIA3 |  |
| NKX2-1 | STARD3NL |  |
| NLGN3 | STARD6 |  |
| NLRP1 | STAT1 |  |
| NMT1 | STAU1 |  |
| NNAT | STC1 |  |
| NOG | STK17B |  |
| NOL4 | STMN1 |  |
| NOVA1 | STRBP |  |
| NPAS3 | STRN3 |  |
| NPEPL1 | STUB1 |  |
| NPTN | STX12 |  |
| NPTX1 | STXBP3 |  |
| NPTX2 | SUB1 |  |
| NR2C2 | SULF1 |  |
| NR3C1 | SULT1A1 |  |
| NR4A2 | SUMO1 |  |
| NR5A2 | SYAP1 |  |
| NRARP | SYCP1 |  |
| NRG2 | SYNE1 |  |
| NRIP1 | SYT1 |  |
| NRIP3 | SYT15 |  |
| NRP1 | TACR3 |  |
| NSD1 | TACSTD2 |  |
| NT5C2 | TAF9 |  |
| NT5C3A | TAF9B |  |
| NUAK2 | TARDBP |  |
| NUDT8 | TBKBP1 |  |
| NUFIP2 | TCEAL8 |  |
| NUMA1 | TCEB1 |  |
| NUS1 | TDP1 |  |
| NABP1 | TDRD7 |  |
| OCLN | TESK2 |  |
| OGFOD2 | TEX264 |  |
| OGT | TFB2M |  |
| ONECUT2 | TFPI |  |
| OSBPL11 | TGFA |  |
| OSBPL6 | TGFBR1 |  |
| OSBPL8 | TGFBR3 |  |
| OSGIN2 | TGM1 |  |
| OTUD4 | TLE3 |  |
| OTUD7B | TLR3 |  |
| OXSR1 | TM7SF3 |  |
| P2RX7 | TMCO1 |  |
| PABPC1 | TMED2 |  |
| PACRG | TMED5 |  |
| PACSIN1 | TMED9 |  |
| PAK2 | TMEM100 |  |
| PAK7 | TMEM106B |  |
| PALM2-AKAP2 | TMEM135 |  |
| PAPD4 | TMEM138 |  |
| PAPD5 | TMEM168 |  |
| PAPD7 | TMEM178A |  |
| PAPPA | TMEM183A |  |
| PAQR9 | TMEM186 |  |
| PARD3B | TMEM218 |  |
| PARG | TMEM33 |  |
| PATL1 | TMEM35 |  |
| PBOV1 | TMEM41B |  |
| PBX1 | TMEM50B |  |
| PBXIP1 | TMEM54 |  |
| PCBP3 | TMEM55A |  |
| PCDH19 | SARAF |  |
| PCDHA1 | TMPO |  |
| PCDHA10 | TMPRSS2 |  |
| PCDHA11 | TMSB4X |  |
| PCDHA12 | TNFAIP1 |  |
| PCDHA13 | TNFRSF18 |  |
| PCDHA2 | TNFSF4 |  |
| PCDHA3 | TNR |  |
| PCDHA4 | TOB1 |  |
| PCDHA5 | TOMM22 |  |
| PCDHA6 | TOX4 |  |
| PCDHA7 | TPBG |  |
| PCDHA8 | TPI1 |  |
| PCDHAC1 | TPM3 |  |
| PCDHAC2 | TPP2 |  |
| PCGF2 | TRAM1 |  |
| PCGF5 | TRAPPC2 |  |
| PCMT1 | TRDN |  |
| PCSK1 | TRHR |  |
| PDCD10 | TRIM32 |  |
| PDE3B | TRIM5 |  |
| PDGFC | TRIP12 |  |
| PDGFRA | TRNT1 |  |
| PDIA3 | TRPC1 |  |
| PDK3 | TRPV5 |  |
| PDK4 | TSHR |  |
| PDPK1 | TSPAN12 |  |
| PDS5A | TSPAN2 |  |
| PDS5B | TSPAN3 |  |
| PDZD3 | TSPAN8 |  |
| PEA15 | TTC12 |  |
| PEAK1 | TTC4 |  |
| PEG3 | TTC9C |  |
| PER3 | TTPA |  |
| PFKFB3 | TMX1 |  |
| PFKFB4 | TXNDC15 |  |
| PGK1 | ERP44 |  |
| PGM2L1 | UBAC1 |  |
| PHACTR2 | UBE2A |  |
| PHC1 | UBE2B |  |
| PHC3 | UBE2D3 |  |
| JADE2 | UBE2F |  |
| JADE1 | UBE2G1 |  |
| PHF20 | UBE2V2 |  |
| PHF21A | UBE4A |  |
| PHF21B | UBLCP1 |  |
| PHF3 | UBQLN1 |  |
| PHF5A | UCHL5 |  |
| PHF6 | UCP3 |  |
| PHIP | UFD1L |  |
| PHKA1 | UFSP2 |  |
| PHLDB1 | UGGT1 |  |
| PHTF2 | UMOD |  |
| PHYHIPL | UQCRQ |  |
| PI15 | USF2 |  |
| PI4K2A | USP1 |  |
| PI4KB | USP14 |  |
| PID1 | USP48 |  |
| PIGA | UTRN |  |
| PIK3R1 | UXS1 |  |
| PIK3R3 | VAMP3 |  |
| PIP4K2B | VCPIP1 |  |
| PITPNA | VDAC1 |  |
| PITPNB | VEZT |  |
| PKP4 | VPS33B |  |
| PLA2G4F | VPS4B |  |
| PLAA | VPS54 |  |
| PLAG1 | VSIG4 |  |
| PLAU | VSNL1 |  |
| PLCB1 | WARS |  |
| PLCD3 | WEE1 |  |
| PLEKHA2 | WFDC1 |  |
| PLEKHH1 | NELFA |  |
| PLXNA2 | WWP1 |  |
| PLXNA4 | XKR4 |  |
| PNISR | XKR8 |  |
| PNPLA6 | XRCC4 |  |
| POLDIP2 | YIPF1 |  |
| POMT2 | YIPF3 |  |
| POU2F1 | YIPF4 |  |
| POU2F2 | YIPF5 |  |
| POU3F2 | YIPF6 |  |
| POU3F4 | YME1L1 |  |
| POU4F2 | YPEL5 |  |
| PPARGC1A | YTHDC1 |  |
| PPARGC1B | YWHAB |  |
| PPIP5K2 | YWHAE |  |
| PPM1B | YWHAH |  |
| PPM1E | YWHAQ |  |
| PPM1K | ZBTB1 |  |
| PPP1CB | ZBTB25 |  |
| PPP1CC | ZBTB44 |  |
| PPP1R10 | ZC3H15 |  |
| PPP1R12A | ZDHHC7 |  |
| PPP1R3D | ZNF414 |  |
| PPP1R9A | ZNF22 |  |
| PPP1R9B | ZFYVE27 |  |
| PPP2R2C | ZHX1 |  |
| PPP2R3A | ZMYND11 |  |
| PPP2R3C | ZNF394 |  |
| PPP3R1 | ZNF655 |  |
| PPP6C | ZNF672 |  |
| PPP6R1 | ZPBP2 |  |
| PPP6R2 |  |  |
| PPT2 |  |  |
| PPTC7 |  |  |
| PRDM1 |  |  |
| PRDM15 |  |  |
| PRDM8 |  |  |
| PRICKLE2 |  |  |
| PRKAA1 |  |  |
| PRKAB2 |  |  |
| PRKAG1 |  |  |
| PRKAG2 |  |  |
| PRKAR1B |  |  |
| PRKCA |  |  |
| PRKCE |  |  |
| PRKG1 |  |  |
| PRMT8 |  |  |
| PRPF19 |  |  |
| PRPF4B |  |  |
| PRR14L |  |  |
| PRR5L |  |  |
| PRRT2 |  |  |
| PSD3 |  |  |
| PSEN1 |  |  |
| PSMA5 |  |  |
| PSME3 |  |  |
| PSME4 |  |  |
| PSMF1 |  |  |
| PSPH |  |  |
| PTAFR |  |  |
| PTCD3 |  |  |
| PTCH1 |  |  |
| PTEN |  |  |
| PTER |  |  |
| PTGES3 |  |  |
| PTGS2 |  |  |
| PTH |  |  |
| PTMA |  |  |
| HACD2 |  |  |
| PTPN14 |  |  |
| PTPRD |  |  |
| PTPRM |  |  |
| PURB |  |  |
| PUS10 |  |  |
| PVRL1 |  |  |
| PWWP2B |  |  |
| QKI |  |  |
| RAB11FIP2 |  |  |
| RAB14 |  |  |
| RAB15 |  |  |
| RAB1B |  |  |
| RAB21 |  |  |
| RAB33B |  |  |
| RAB34 |  |  |
| RAB35 |  |  |
| RAB6A |  |  |
| RABGAP1 |  |  |
| RAD23B |  |  |
| RAD51D |  |  |
| RAI1 |  |  |
| RAI14 |  |  |
| RALA |  |  |
| RALBP1 |  |  |
| RALGPS1 |  |  |
| RANBP10 |  |  |
| RANBP3 |  |  |
| RAP1B |  |  |
| RAP2C |  |  |
| RAPGEF5 |  |  |
| RASA3 |  |  |
| RASAL1 |  |  |
| RASAL2 |  |  |
| RASGEF1A |  |  |
| RASL12 |  |  |
| RASSF5 |  |  |
| RASSF8 |  |  |
| RBFOX1 |  |  |
| RBFOX2 |  |  |
| RBM24 |  |  |
| RBM26 |  |  |
| RBM38 |  |  |
| RBM4 |  |  |
| RC3H1 |  |  |
| RCC2 |  |  |
| RCN1 |  |  |
| RCOR1 |  |  |
| RDH10 |  |  |
| REEP3 |  |  |
| REG4 |  |  |
| REPS2 |  |  |
| RER1 |  |  |
| RERE |  |  |
| REST |  |  |
| REV1 |  |  |
| RFFL |  |  |
| RFWD2 |  |  |
| RFX7 |  |  |
| RGAG1 |  |  |
| RGS22 |  |  |
| RGS4 |  |  |
| RGS5 |  |  |
| RHBDF2 |  |  |
| RHOBTB2 |  |  |
| RICTOR |  |  |
| RIMBP2 |  |  |
| RIMS2 |  |  |
| RIMS3 |  |  |
| RIOK3 |  |  |
| RLIM |  |  |
| RMND5A |  |  |
| RNF11 |  |  |
| RNF122 |  |  |
| RNF125 |  |  |
| RNF138 |  |  |
| RNF144A |  |  |
| RNF145 |  |  |
| RNF165 |  |  |
| RNF24 |  |  |
| RNF34 |  |  |
| RNF38 |  |  |
| RNF44 |  |  |
| RNF6 |  |  |
| ROBO1 |  |  |
| ROBO2 |  |  |
| ROCK1 |  |  |
| PTBP3 |  |  |
| RORA |  |  |
| RPGRIP1L |  |  |
| RPH3AL |  |  |
| RPL36A-HNRNPH2 |  |  |
| RPRD1A |  |  |
| RPS6KA3 |  |  |
| RPS6KA6 |  |  |
| RPS6KB1 |  |  |
| RPS6KL1 |  |  |
| RSBN1L |  |  |
| RSPO3 |  |  |
| RSRC2 |  |  |
| RTN3 |  |  |
| RUNX1T1 |  |  |
| RXFP2 |  |  |
| S100PBP |  |  |
| S1PR1 |  |  |
| SACM1L |  |  |
| SALL1 |  |  |
| SAMD12 |  |  |
| SAMD4A |  |  |
| SAMD8 |  |  |
| SASH1 |  |  |
| SATB1 |  |  |
| SATB2 |  |  |
| SBF2 |  |  |
| SBK1 |  |  |
| SBNO1 |  |  |
| SCAF11 |  |  |
| SCAF8 |  |  |
| SCAMP1 |  |  |
| SCAMP4 |  |  |
| SCARA3 |  |  |
| SCGB1A1 |  |  |
| SCN2A |  |  |
| SDCBP |  |  |
| SEC22A |  |  |
| SEC62 |  |  |
| SEMA3A |  |  |
| SEMA6A |  |  |
| SENP5 |  |  |
| SENP6 |  |  |
| SEPN1 |  |  |
| SERAC1 |  |  |
| SERINC3 |  |  |
| SERINC5 |  |  |
| SERP1 |  |  |
| SERPINE1 |  |  |
| SERTAD2 |  |  |
| SESTD1 |  |  |
| SETD1B |  |  |
| SETD7 |  |  |
| SETD8 |  |  |
| SGCB |  |  |
| SGMS2 |  |  |
| SGPL1 |  |  |
| SH2B3 |  |  |
| SH3BGRL2 |  |  |
| SH3BP4 |  |  |
| SH3BP5 |  |  |
| SH3KBP1 |  |  |
| SH3PXD2A |  |  |
| SH3RF1 |  |  |
| SH3TC2 |  |  |
| SHANK2 |  |  |
| SHC3 |  |  |
| SHE |  |  |
| SHISA9 |  |  |
| SIDT1 |  |  |
| SIK1 |  |  |
| SIK2 |  |  |
| SIPA1L2 |  |  |
| SIPA1L3 |  |  |
| SIRT7 |  |  |
| SIX4 |  |  |
| SKP1 |  |  |
| SLAIN2 |  |  |
| SLC12A5 |  |  |
| SLC12A6 |  |  |
| SLC13A2 |  |  |
| SLC16A2 |  |  |
| SLC16A6 |  |  |
| SLC17A8 |  |  |
| SLC1A2 |  |  |
| SLC22A23 |  |  |
| SLC24A2 |  |  |
| SLC24A4 |  |  |
| SLC25A44 |  |  |
| SLC26A7 |  |  |
| SLC28A3 |  |  |
| SLC2A1 |  |  |
| SLC2A3 |  |  |
| SLC31A1 |  |  |
| SLC35A3 |  |  |
| SLC35A4 |  |  |
| SLC35B4 |  |  |
| SLC35F1 |  |  |
| SLC38A2 |  |  |
| SLC39A10 |  |  |
| SLC45A4 |  |  |
| SLC4A4 |  |  |
| SLC5A3 |  |  |
| SLC6A14 |  |  |
| SLC6A6 |  |  |
| SLC7A11 |  |  |
| SLC7A2 |  |  |
| SLC8A2 |  |  |
| SLC9A2 |  |  |
| SLITRK1 |  |  |
| SLITRK3 |  |  |
| SLITRK4 |  |  |
| SLN |  |  |
| SMAD2 |  |  |
| SMAD5 |  |  |
| SMARCA5 |  |  |
| SMARCD1 |  |  |
| SMARCD2 |  |  |
| SMC2 |  |  |
| SMEK1 |  |  |
| SMNDC1 |  |  |
| SMPD3 |  |  |
| SNN |  |  |
| SNPH |  |  |
| SNRK |  |  |
| SNX13 |  |  |
| SNX27 |  |  |
| CAPN15 |  |  |
| SORT1 |  |  |
| SOS1 |  |  |
| SOS2 |  |  |
| SOX11 |  |  |
| SOX12 |  |  |
| SOX5 |  |  |
| SOX6 |  |  |
| SP1 |  |  |
| SP3 |  |  |
| SP4 |  |  |
| SPATA5 |  |  |
| SPATS2L |  |  |
| SPCS2 |  |  |
| SPG11 |  |  |
| SPRY3 |  |  |
| SPRY4 |  |  |
| SPRYD3 |  |  |
| SPSB4 |  |  |
| SPTBN4 |  |  |
| SREBF2 |  |  |
| SRF |  |  |
| SRGAP1 |  |  |
| SRPK1 |  |  |
| SRPK2 |  |  |
| SRSF1 |  |  |
| SRSF10 |  |  |
| SRSF11 |  |  |
| SRSF6 |  |  |
| SSH2 |  |  |
| SSR1 |  |  |
| SSR3 |  |  |
| ST8SIA3 |  |  |
| ST8SIA4 |  |  |
| STAG2 |  |  |
| STAG3L4 |  |  |
| STAM |  |  |
| STARD13 |  |  |
| STARD8 |  |  |
| STC2 |  |  |
| STK11 |  |  |
| STK17B |  |  |
| STK39 |  |  |
| STMN3 |  |  |
| STRADB |  |  |
| STRN |  |  |
| STRN3 |  |  |
| STT3A |  |  |
| STX3 |  |  |
| STXBP6 |  |  |
| STYX |  |  |
| SUFU |  |  |
| SULF1 |  |  |
| SUN2 |  |  |
| SVEP1 |  |  |
| SVIL |  |  |
| SVOP |  |  |
| SYAP1 |  |  |
| SYCP1 |  |  |
| SYDE2 |  |  |
| SYNCRIP |  |  |
| SYNGAP1 |  |  |
| SYNJ1 |  |  |
| SYT1 |  |  |
| SYT10 |  |  |
| SYT11 |  |  |
| SYT2 |  |  |
| SYT3 |  |  |
| SYT4 |  |  |
| SYT6 |  |  |
| SYT9 |  |  |
| TANC2 |  |  |
| TAOK1 |  |  |
| TAOK2 |  |  |
| TAPBP |  |  |
| TARDBP |  |  |
| TAX1BP1 |  |  |
| TBC1D12 |  |  |
| TBC1D15 |  |  |
| TBCEL |  |  |
| TBKBP1 |  |  |
| TBL1XR1 |  |  |
| TBPL1 |  |  |
| TCF20 |  |  |
| TCF7L1 |  |  |
| TCP11L1 |  |  |
| TEAD1 |  |  |
| TET2 |  |  |
| TF |  |  |
| TFAP2A |  |  |
| TFAP2B |  |  |
| TFCP2L1 |  |  |
| TFRC |  |  |
| TGFA |  |  |
| TGFB2 |  |  |
| TGFBR2 |  |  |
| TGFBR3 |  |  |
| TGIF2 |  |  |
| THAP2 |  |  |
| THRB |  |  |
| THY1 |  |  |
| TICAM2 |  |  |
| TIMM17B |  |  |
| TIPARP |  |  |
| TJP1 |  |  |
| TLE4 |  |  |
| TLX3 |  |  |
| TM9SF3 |  |  |
| TMBIM6 |  |  |
| TMCO1 |  |  |
| TMED7 |  |  |
| TMED7-TICAM2 |  |  |
| TMEM121 |  |  |
| TMEM127 |  |  |
| TMEM151B |  |  |
| TMEM170B |  |  |
| TMEM180 |  |  |
| TMEM183A |  |  |
| TMEM183B |  |  |
| TMEM184A |  |  |
| SLC35G1 |  |  |
| TMEM201 |  |  |
| TMEM203 |  |  |
| TMEM207 |  |  |
| TMEM25 |  |  |
| TMEM33 |  |  |
| TMEM35 |  |  |
| TMEM41B |  |  |
| TMEM52 |  |  |
| TMEM54 |  |  |
| TMEM63B |  |  |
| TMEM9B |  |  |
| TMF1 |  |  |
| TMOD1 |  |  |
| TMSB10 |  |  |
| TMTC2 |  |  |
| TNFAIP1 |  |  |
| TNFRSF19 |  |  |
| TNKS2 |  |  |
| TNP1 |  |  |
| TNPO1 |  |  |
| TNPO3 |  |  |
| TNRC6A |  |  |
| TNRC6B |  |  |
| TNS3 |  |  |
| TNXB |  |  |
| TOB1 |  |  |
| TOMM70A |  |  |
| TPD52 |  |  |
| TPM3 |  |  |
| TPM4 |  |  |
| TPR |  |  |
| TRA2B |  |  |
| TRAF3 |  |  |
| TRAK1 |  |  |
| TRAK2 |  |  |
| TRAPPC8 |  |  |
| TRIM25 |  |  |
| TRIM26 |  |  |
| TRIM3 |  |  |
| TRIM54 |  |  |
| TRIM67 |  |  |
| TRPC1 |  |  |
| TRPS1 |  |  |
| TRPV3 |  |  |
| TSC22D2 |  |  |
| TSC22D3 |  |  |
| CEP41 |  |  |
| TSKU |  |  |
| TSN |  |  |
| TSPAN3 |  |  |
| TSPAN5 |  |  |
| TSPYL1 |  |  |
| TTC13 |  |  |
| TTC39A |  |  |
| TTLL4 |  |  |
| TTPA |  |  |
| TTYH3 |  |  |
| TULP4 |  |  |
| TWF1 |  |  |
| TXNIP |  |  |
| UBAC1 |  |  |
| UBE2B |  |  |
| UBE2D1 |  |  |
| UBE2D3 |  |  |
| UBE2E3 |  |  |
| UBE2J1 |  |  |
| UBE2K |  |  |
| UBE2O |  |  |
| UBE2Q1 |  |  |
| UBE2R2 |  |  |
| UBE2W |  |  |
| UBE3A |  |  |
| UBFD1 |  |  |
| UBP1 |  |  |
| UBR2 |  |  |
| UBR3 |  |  |
| UBTF |  |  |
| UBXN1 |  |  |
| UCK2 |  |  |
| UCP3 |  |  |
| UGGT1 |  |  |
| UHMK1 |  |  |
| ULK3 |  |  |
| UMOD |  |  |
| UNC5A |  |  |
| UNC5C |  |  |
| UNC80 |  |  |
| UPF1 |  |  |
| USP14 |  |  |
| USP32 |  |  |
| USP33 |  |  |
| USP42 |  |  |
| USP45 |  |  |
| USP47 |  |  |
| USP48 |  |  |
| USP6 |  |  |
| USP6NL |  |  |
| USP7 |  |  |
| UXS1 |  |  |
| VAMP1 |  |  |
| VAMP4 |  |  |
| VAMP7 |  |  |
| VANGL1 |  |  |
| VAPB |  |  |
| VASH1 |  |  |
| VASH2 |  |  |
| VAV2 |  |  |
| VCAN |  |  |
| VCL |  |  |
| VEGFA |  |  |
| VIT |  |  |
| VOPP1 |  |  |
| VPS13C |  |  |
| VPS26B |  |  |
| VPS37A |  |  |
| VPS4A |  |  |
| VPS54 |  |  |
| VSIG4 |  |  |
| VTA1 |  |  |
| WASF2 |  |  |
| WDR20 |  |  |
| WDR26 |  |  |
| WDR37 |  |  |
| WDR43 |  |  |
| WDR44 |  |  |
| WHSC1 |  |  |
| WIPF1 |  |  |
| WNK1 |  |  |
| WNT1 |  |  |
| WNT10B |  |  |
| WNT3A |  |  |
| WNT5A |  |  |
| WTAP |  |  |
| WTIP |  |  |
| WWC3 |  |  |
| WWP1 |  |  |
| XIAP |  |  |
| XPNPEP3 |  |  |
| XPO4 |  |  |
| XYLT1 |  |  |
| YAF2 |  |  |
| YIF1B |  |  |
| YPEL2 |  |  |
| YPEL3 |  |  |
| YTHDC1 |  |  |
| YWHAB |  |  |
| YWHAG |  |  |
| YWHAH |  |  |
| YY1 |  |  |
| ZBED4 |  |  |
| ZBED5 |  |  |
| ZBTB10 |  |  |
| ZBTB20 |  |  |
| ZBTB34 |  |  |
| ZBTB39 |  |  |
| ZBTB44 |  |  |
| ZBTB7A |  |  |
| ZBTB8A |  |  |
| ZC3H12B |  |  |
| ZC3H12C |  |  |
| ZC3H12D |  |  |
| ZC3H7B |  |  |
| ZC3HAV1 |  |  |
| ZCCHC11 |  |  |
| ZCCHC14 |  |  |
| ZCCHC2 |  |  |
| ZDBF2 |  |  |
| ZDHHC15 |  |  |
| ZDHHC17 |  |  |
| ZDHHC3 |  |  |
| ZDHHC5 |  |  |
| ZDHHC7 |  |  |
| ZEB1 |  |  |
| ZFAND3 |  |  |
| ZFHX3 |  |  |
| ZFHX4 |  |  |
| ZFP36L1 |  |  |
| ZFP36L2 |  |  |
| ZFP91 |  |  |
| ZFPM2 |  |  |
| ZFYVE26 |  |  |
| ZHX3 |  |  |
| ZIC5 |  |  |
| ZKSCAN1 |  |  |
| ZMAT3 |  |  |
| ZMIZ1 |  |  |
| ZMYM2 |  |  |
| ZNF148 |  |  |
| ZNF217 |  |  |
| ZBTB18 |  |  |
| ZNF275 |  |  |
| ZNF280D |  |  |
| ZNF282 |  |  |
| ZNF329 |  |  |
| ZNF385A |  |  |
| ZNF395 |  |  |
| ZNF423 |  |  |
| ZNF469 |  |  |
| ZNF608 |  |  |
| ZNF618 |  |  |
| ZNF652 |  |  |
| ZNF654 |  |  |
| ZNF687 |  |  |
| ZNF704 |  |  |
| ZNF710 |  |  |
| ZNF711 |  |  |
| ZNF770 |  |  |
| ZNF784 |  |  |
| ZNF831 |  |  |
| ZNFX1 |  |  |
| ZNRF2 |  |  |
| ZNRF3 |  |  |
| ZRANB2 |  |  |
| ZSCAN20 |  |  |
| ZYX |  |  |
| ZZZ3 |  |  |
